# Macrophage repolarization by immune checkpoint blockade drives T cell engagement in the tumor microenvironment

**DOI:** 10.1016/j.isci.2025.113538

**Published:** 2025-09-10

**Authors:** Tina Kwok, Ildefonso A. Silva-Junior, Sara Korpe, Haidong Dong, Jessica N. Lancaster

**Affiliations:** 1 Department of Immunology, Mayo Clinic, Phoenix, AZ 85054, USA; 2 Department of Immunology, Mayo Clinic, Rochester, MN 55905, USA; 3 Department of Cancer Biology, Mayo Clinic, Phoenix, AZ 85054, USA

**Keywords:** Microenvironment, Immunology, Cancer

## Abstract

Immunotherapy combinations can improve patient outcomes, yet the interactions within the tumor microenvironment (TME) that drive therapeutic synergy are poorly understood. Tumor establishment drives monocyte recruitment and differentiation into tumor-associated macrophages (TAMs), which have essential roles in coordinating immune responses and are thus attractive targets for therapeutic modulation. In a murine model of combination anti-programmed cell death protein 1 (PD-1) and its ligand (anti-PD-L1) checkpoint blockade, tumor control was associated with increased infiltration of CD8^+^ T cells and M1-like repolarization of TAMs. Live-cell imaging of the tumor microenvironment revealed close contacts between tumor-infiltrating CD8^+^ T cells and TAMs, in which the extent of the contact interfaces increased with combination immunotherapy. Treatment with anti-PD-L1 was able to increase macrophage expression of pro-inflammatory factors and phagocytic activity, suggesting a role for TAMs in reactivating CD8^+^ T cells in the TME. However, co-treatment with anti-PD-1 was ultimately necessary for tumor control, indicating the need for combination targeting of the TME.

## Highlights

• Tumor-associated macrophages (TAMs) make direct contacts with CD8^+^ T cells
• Checkpoint blockade increases contact surface area between TAMs and T cells
• Macrophage repolarization via anti-PD-L1 requires macrophage PD-L1 expression
• Combination anti-PD-1 and anti-PD-L1 enhances TAM repolarization and tumor control

## Graphical abstract

**Figure:**
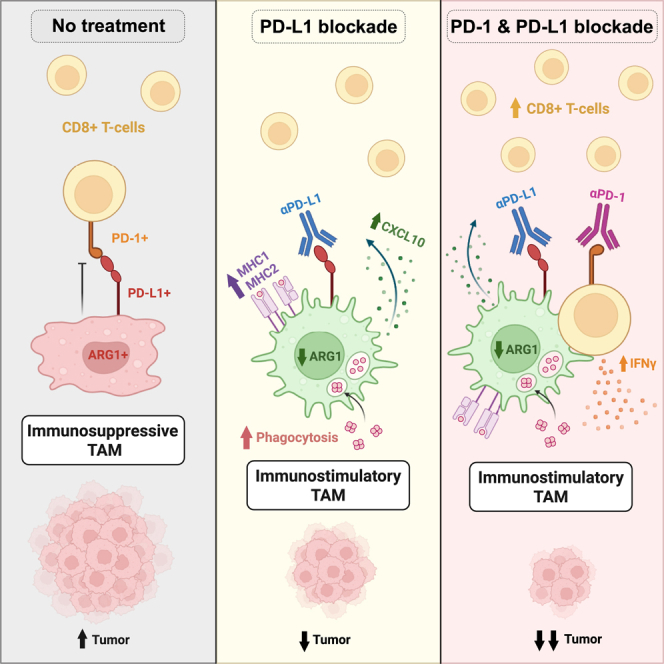

## Introduction

Immune checkpoint blockade (ICB) therapies, such as those targeting programmed cell death protein 1 (PD-1) and its ligand PD-L1, have shown promising clinical potential in treating various cancers^1^; however, only ∼30% of patients with solid tumors experience improved responses.^2^ PD-1 is an inhibitory receptor expressed on activated T cells,^3^^,^^4^ and its binding to PD-L1 on tumor or other immune cells results in dampened T cell activation.^5^ Within the context of the tumor microenvironment (TME), the efficacy of ICB therapies relies on the infiltration of CD8^+^ T cells^6^ and their effective immunostimulatory interactions with antigen-presenting cells (APCs).^7^ Despite the capacity of ICB therapies to robustly enhance T cell function,^8^^,^^9^ the parameters within the TME that define ICB therapeutic efficacy remain poorly understood.

Clinical data have revealed the importance of the myeloid immune compartment in therapeutic resistance.^10^ Myeloid lineage cells such as tumor-associated macrophages (TAMs) constitute the majority of immune-infiltrating cells in the tumor,^11^ and their abundance is often associated with poor prognosis in human cancers. Antigenic shedding and inflammatory signaling originating from the tumor initially recruit monocytes from blood circulation.^12^ Heavily influenced by the local microenvironment, they adopt an immunosuppressive phenotype in the tumor, in which they promote tumor progression and suppress T cell activity.^12^ These immunosuppressive TAMs are often identified by their expression of arginase 1 (ARG1),^13^ an enzyme that functions to deplete arginine, which is essential for T cell activation and proliferation.^14^ Previous research has shown that TAMs can sequester anti-PD-1 off T cells,^15^ inhibit CD8^+^ T cell infiltration into tumor nests,^16^ and contribute to T cell exhaustion.^17^^,^^18^ Thus, targeting myeloid cells has gained significant traction as a therapeutic strategy.^19^ However, clinical trials that have targeted known signaling axes for myeloid cell survival, such as CSF1R, or monocyte recruitment, such as CCR2, have had nominal success.^19^ The limited clinical benefit of myeloid cell depletion suggests that TAMs, while part of the immunosuppressive TME, must also have a role in therapeutic efficacy.

Macrophages are highly plastic^14^ and heterogeneous in nature, and recent studies suggest that macrophages can be reprogrammed toward a pro-inflammatory phenotype.^20^ Macrophages have been reported to express PD-1 and PD-L1 in a context-dependent manner, suggesting that they may respond to ICB. It has been shown that macrophage proliferation and survival,^21^ as well as their secretion of effector molecules such as interferon-gamma (IFNγ) *in vitro*,^22^ could be improved by treatment with anti-PD-L1. Preclinical studies in immunocompetent mice have demonstrated that tumor expression of PD-L1 was dispensable for anti-PD-L1 ICB efficacy.^23^ Rather, experiments and clinical data indicated that myeloid PD-L1 expression predicted PD-1/PD-L1 blockade outcomes.^3^ More recently, the immunoengineered cytokine PD1-IL2v was shown to synergize with anti-PD-L1 reprogramming of the TAM compartment.^24^ Meta-analyses across multiple cancers and ICB modalities has revealed strong interactions between ICB responsiveness with T cell and myeloid cell immune signatures.^25^ Experiments in mice demonstrated that PD-1 blockade can modulate TAMs, which produce ligands for the chemokine receptor CXCR3 upregulated on activated CD8^+^ T cells, thus improving T cell infiltration.^26^^,^^27^^,^^28^^,^^29^ Taken together, these findings support TAM repolarization during ICB. While progress has been made in establishing TAM reprogramming as a viable therapeutic approach, the mechanisms by which ICB induces TAMs to support anti-tumor immunity are still incomplete. Namely, it is important to define the signals upstream of T cell reactivation in the TME and whether this is enhanced by direct antigen presentation, costimulatory signaling, and/or cytokine milieu.^30^

To address this, we investigated the cellular mechanisms by which TAMs promote T cell anti-tumor activity within the TME during ICB. Live-cell imaging revealed intimate contacts between TAMs and CD8^+^ T cells within tumors of mice treated by anti-PD-1 and anti-PD-L1 antibodies. Our experiments further support the capacity for anti-PD-L1 treatment to directly elicit a proinflammatory phenotype in tumor macrophages, which was dependent on macrophage expression of PD-L1. We observed significant downregulation of ARG1 expression, increased expression of antigen presentation molecules and the T cell-recruiting chemokine CXCL10, as well as increased phagocytic capacity. Furthermore, anti-PD-L1-treated macrophages exhibited improved capacity for antigen-specific stimulation of CD8^+^ T cells, which was cell contact dependent. Our findings support a model in which TAM responsiveness to ICB promotes direct T cell engagement in a myeloid PD-L1-specific manner.

## Results

### TAMs within checkpoint antibody-treated tumors make wide contact interfaces with infiltrating CD8^+^ T cells

**Figure 1:**
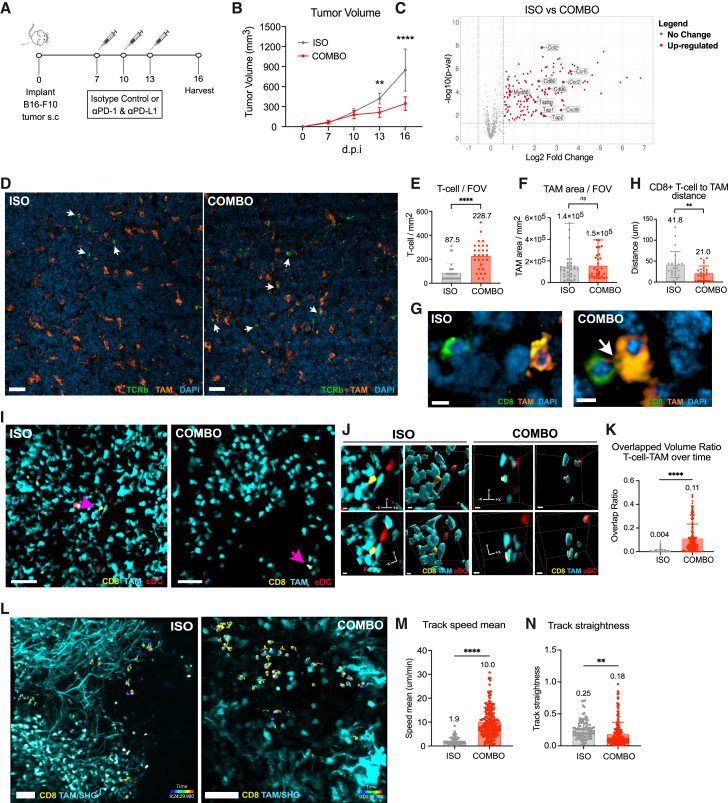
TAMs within checkpoint antibody-treated tumors make wide contact interfaces with infiltrating CD8^+^ T cells (A) Experimental schematic of subcutaneous B16-F10 melanoma tumor mouse model treated with 3 doses of 200 μg of IgG2A isotype control or combination 200 μg anti-PD-1 and 200 μg anti-PD-L1 antibody intraperitoneally and harvested on day 16. (B) Tumor growth curves of isotype control and combo-treated mice. Data shown represent combined data from 3 experiments with *n* = 3 mice per experimental group. (C) Volcano plot of transcriptomic differences of total tumor cells isolated from isotype control and combo-treated B16-F10 tumors. Gray indicates no significant change, and red indicates differentially upregulated genes (148 genes). (D) Representative images of isotype control and combo-treated B16-F10 tumor cryosections stained with antibodies against TCRβ (green), F4/80 (orange), and DAPI (blue). Scale bars represent 50 μm; arrows denote T cells. (E) Quantification of mean T cell count. (F) TAM area in fields of view. Data shown represent combined data from 3 to 4. experiments, 2 tumors per experimental group; number of cells: Iso = 29, Combo = 28. Data displayed as means ± SEMs. (G) Representative images of CD8α (green)-, F4/80 (orange)-, and DAPI (blue)-stained tumor cryosections from isotype control and combo-treated tumors. Scale bars represent 5 μm. (H) Quantification of mean CD8^+^ T cell to TAM distance. Direct interaction characterized by < 3 μm distance. Data shown represent combined data from 3 experiments, 2 tumors per experimental group; number of cells: Iso = 23, Combo = 25. Data displayed as means ± SEMs. (I) Live two-photon imaging of isotype control and combo-treated LLC tumors from MacBlue;*CD11c*-mCherry mice with adoptively transferred EGFP-CD8^+^ T cells (yellow). CD8^+^ T cell and TAM interactions denoted by magenta arrows. Scale bars represent 50 μm. (J) Zoomed in two-photon imaging of (I) with 2 different angles. Scale bars represent 5 μm. (K) Quantification of CD8^+^ T cell to TAM overlapped volume ratio over time in isotype control and combo-treated tumors at 15 days post-inoculation (dpi). Data shown represent combined data from 3 experiments, 2 tumors per experimental group; number of cells: Iso = 7, Combo = 7, at 40 time points. (L) Live two-photon imaging of isotype control and combo-treated LLC tumors from MacBlue or MAFIA mice with adoptively transferred dsRed-CD8^+^ T cells (yellow). Macrophages and second harmonic generation displayed in blue. T cell migration tracks displayed as multicolored lines. Scale bars represent 50 μm. (M) Quantification of CD8^+^ T cell track speed mean in isotype control and combo-treated tumors at 15 dpi. Data shown represent combined data from 3 to 4 experiments, 2 tumors per experimental group; number of cells: Iso = 96, Combo = 184. Data displayed as means ± SEMs. (N) Quantification of CD8^+^ T cell track straightness in isotype control and combo-treated tumors at 15 dpi. Data displayed as means ± SEMs. Analyzed by Student’s t test, one- or two-way ANOVA; ∗∗*p* <0.01, ∗∗∗∗*p* <0.0001.

We determined that subcutaneously implanted B16-F10 melanoma tumors within C57BL/6J mice grew slower when 3 doses of combined anti-PD-1 and anti-PD-L1 antibodies were administered as compared to isotype antibody-treated controls (Figures 1A and 1B). NanoString RNA sequencing of dissociated tumors at endpoint revealed 148 differentially upregulated genes following combination antibody treatment (Figure 1C). Notably, multiple genes involved in antigen presentation (*Tap1/2, B2m*) and nuclear factor κB (NF-κB) signaling (*Myd88*) were upregulated upon combination treatment, indicating an activated and pro-inflammatory TME (Figure S1A).^31^^,^^32^ In addition, genes involved in chemokine signaling (*Ccl2, Cxcl9*) were upregulated following treatment, suggesting improved immune cell recruitment to the tumor (Figure S1A).^33^ By immunofluorescence, T cell densities were increased with treatment, although TAM densities remained constant (Figures 1D–1F). CD8^+^ T cell staining revealed that combination treatment decreased the distance between CD8^+^ T cells and TAMs, resulting in 28% of the CD8^+^ T cells engaging in direct interactions with macrophages (Figures 1G and 1H).

For live-cell imaging of myeloid cell interactions with infiltrating CD8^+^ T cells, we adoptively transferred EGFP+ CD8^+^ T cells into MacBlue;*CD11c*-mCherry recipients 24 h prior to Lewis lung carcinoma (LLC) tumor implantation. In these fluorescent reporter mice, *Csf1r* drives a blue fluorescence signal in monocytes and macrophages and CD11c+ conventional dendritic cells express red fluorescence, allowing the visualization of macrophages and dendritic cells that have been recruited to the tumor. Similar to B16-F10 tumors, LLC tumors treated with a combination of anti-PD-1 and anti-PD-L1 displayed significantly reduced tumor growth rate compared to isotype control-treated mice (Figure S1B). For live-cell two-photon microscopy, excised tumors were embedded in low-melting-point agarose and vibratome sectioned into 300- to 400-μm-thick tissue slices.^34^ Imaging the slices within a warmed chamber that continuously circulated fresh, oxygenated medium, we observed that tumor-infiltrating EGFP+ CD8^+^ T cells were rare and in proximity to the abundant MacBlue+ macrophages (Figures 1I and 1J, Videos S1, S2, S3, and S4). Two-photon *ex vivo* imaging of treated tumor sections revealed that CD8^+^ T cells and TAMs in combination antibody-treated tumors were in closer proximity and displayed a greater extent of engagement than those in isotype-treated tumors, as quantified by cell surface area overlap (Figure 1K). Because spectral overlap between MacBlue (*Csf1r*-ECFP) and EGFP required extensive unmixing, we conducted subsequent imaging experiments using dsRed+ CD8^+^ T cells adoptively transferred into MacBlue^+^;*CD11c*-mCherry^-^ or *Csf1r*-EGFP mice to improve spectral separation of T cell and macrophage signals. CD8^+^ T cells in treated tumor slices displayed higher motility and high confinement within the tumors, with average speed of 10 μm min^−1^ and track straightness of 0.18, as compared to isotype control tumors (1.9 μm min^−1^ and 0.25, respectively) (Figures 1L–1N and S1C and Videos S5 and S6). As a comparison, EGFP+ CD8^+^ naive T cells transferred into naive MacBlue mice had average speeds of 24 μm min^−1^ and track straightness of 0.46 in the lymph nodes, as expected^35^ (Figure S1D, Video S7). Thus, we observed that CD8^+^ T cells have increased surface engagement with TAMs during ICB treatment, which was accompanied by increased cell speeds and path confinements.

Video S1. CD8+ T cell and TAM interactions in tumor treated with isotype control antibody, related to Figure 1I Live two-photon imaging of isotype control-treated LLC tumor slices with adoptively transferred EGFP-CD8^+^ T cells (yellow), TAMs (blue), and cDCs (red). Images were acquired for 15–20 min with 20-s time intervals, through a depth of 40 μm, and a maximum intensity projection is displayed. Scale bar represents 50 μm.

Video S2. CD8+ T cell and TAM interactions in tumor treated with combo anti-PD1/PD-L1 antibody, related to Figure 1I Live two-photon imaging of combo-treated LLC tumor slices with adoptively transferred EGFP-CD8^+^ T cells (yellow), TAMs (blue), and cDCs (red). Images were acquired for 15–20 min with 20-s time intervals, through a depth of 40 μm, and a maximum intensity projection is displayed. Scale bar represents 50 μm.

Video S3. CD8+ T cell and TAM interactions in tumor treated with isotype control antibody, related to Figure 1J Zoomed in version of Video S1. Scale bar represents 5 μm.

Video S4. CD8+ T cell and TAM interactions in tumor treated with combo anti-PD1/PD-L1 antibody, related to Figure 1J Zoomed in version of Video S2. Scale bar represents 5 μm.

Video S5. CD8+ T cell tracks in tumor treated with isotype control antibody, related to Figure 1L Live two-photon imaging of isotype control-treated LLC tumor slices with adoptively transferred dsRed-CD8^+^ T cells (yellow), TAMs (blue), and SHG (blue). Images were acquired for 20–25 min with 20-s time intervals, through a depth of 40 μm, and a maximum intensity projection is displayed. Tracks are color encoded for time. Scale bar represents 50 μm.

Video S6. CD8+ T cell tracks in tumor treated with combo anti-PD1/PD-L1 antibodies, related to Figure 1L Live two-photon imaging of combo-treated LLC tumor slices with adoptively transferred dsRed-CD8^+^ T cells (yellow), TAMs (blue), and SHG (blue). Images were acquired for 20–25 min with 20-s time intervals, through a depth of 40 μm, and a maximum intensity projection is displayed. Tracks are color encoded for time. Scale bar represents 50 μm.

Video S7. Naive CD8+ T cell tracks in lymph node section, related to Figure S1D Live two-photon imaging of GFP+ CD8^+^ T cells (green) and macrophages (blue) in naive mouse lymph node slices. Images were acquired for 15–20 min with 20-s time intervals, through a depth of 40 μm, and a maximum intensity projection is displayed. Tracks are color encoded for time. Scale bar represents 50 μm.

### TAMs within checkpoint antibody-treated tumors appear repolarized and reshape their morphology

**Figure 2:**
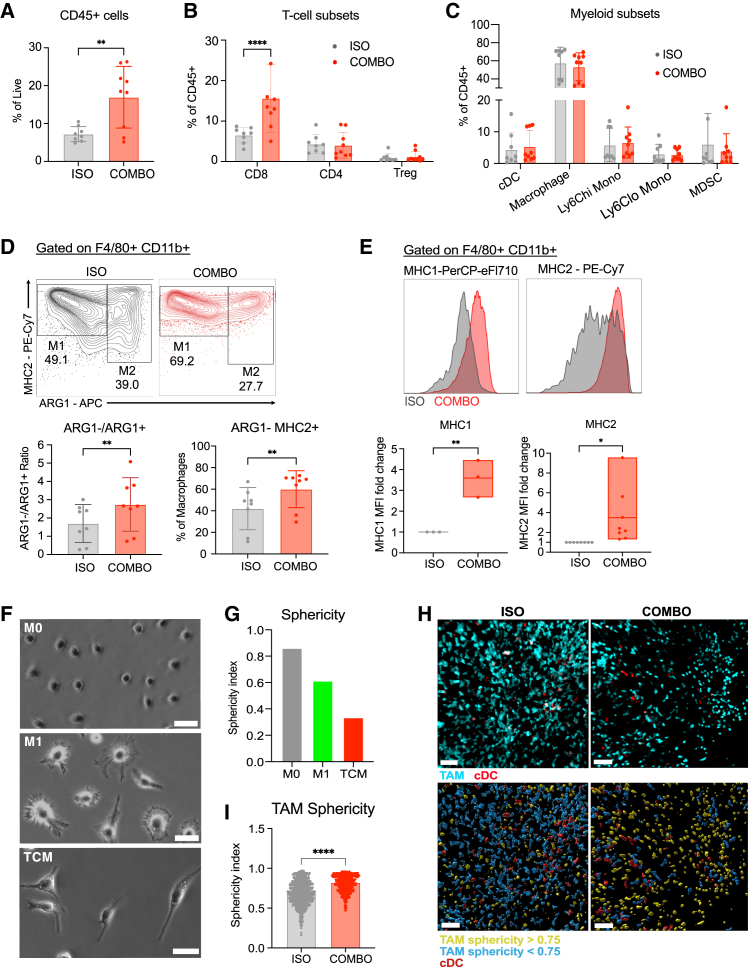
TAMs within checkpoint antibody-treated tumors appear repolarized and reshape their morphology (A) Flow cytometric quantification of CD45^+^ immune cell infiltrate in isotype control and combo-treated tumors at 16 dpi. Data shown represent combined data from 3 independent experiments with *n* = 3 mice per group. Each symbol represents a single tumor, and data are displayed as means ± SEMs. (B) Frequencies of T cell subsets of CD45^+^ cells in isotype control and combo-treated tumors at 16 dpi. (C) Frequencies of myeloid immune cell subsets of CD45^+^ cells in isotype control and combo-treated tumors at 16 dpi. (D) Representative flow cytometry plots of ARG1 and MHC2 within F4/80+CD11b+ TAMs (top panel). Ratio of ARG1−/ARG1+ within TAM population (bottom left panel). Frequency of ARG1− MHC2+ TAMs (bottom right panel). (E) Histograms and mean fluorescence intensities (MFI) of MHC1 and MHC2 within F4/80+CD11b+ TAMs in isotype control and combo-treated mice at 16 dpi. (F) Representative bright-field microscopic imaging of M0, M1, and TCM cells. Scale bars represent 30 μm. (G) Representative mean sphericity of M0, M1, and TCM cells. (H) Representative images of tumor slices at 15 dpi from CSF1R-MacBlue and CD11c-mCherry reporter mice (top panels). Surface masks of yellow-highlighted cells indicate TAM sphericity >0.75, blue indicates TAM sphericity <0.75, and red indicates conventional DC cells (bottom panels). Scale bars represent 50 μm. (I) Quantification of TAM sphericity index from isotype control and combo-treated LLC tumors. Data collected from 3 to 4 experiments, 2 tumors per experimental group; number of cells: Iso = 842, Combo = 701. Data displayed as means ± SEMs. Analyzed by Student’s t test, one-way or two-way ANOVA; ∗*p* <0.05, ∗∗ *p* <0.01, ∗∗∗∗ *p* <0.0001.

Flow cytometric analyses confirmed increased overall CD45^+^ immune cell infiltration and CD8^+^ T cell abundance following treatment (Figures 2A and 2B, gating in Figure S2A). Although frequencies of myeloid subsets were similar between the groups (Figure 2C), a decreased proportion of ARG-1+ putative M2-like macrophages were observed with treatment, leading to increased ratios of ARG-1- to ARG-1+ TAMs and increased frequencies of ARG1- MHC2+ TAMs (Figure 2D). Macrophages in treated tumors expressed significantly higher levels of antigen presentation molecules major histocompatibility complexes I (MHC1) and II (MHC2) (Figure 2E). Thus, in a mouse model of immune checkpoint-responsive tumors, we found that increased CD8^+^ T cell infiltration was associated with changes in TAM expression of ARG1, MHC1, and MHC2.

To model TAMs *in vitro*, we adapted a protocol^36^ to generate tumor-conditioned macrophages (TCMs). Here, bone marrow cells isolated from C57BL/6J mice were differentiated using macrophage colony stimulating factor into macrophages (M0) before polarization toward an M2-like, TCM phenotype by supplementation with interleukin (IL)-4, IL-10, and B16-F10 tumor-conditioned media. For comparison, M0 cells were polarized toward the M1 phenotype by adding IFNγ and lipopolysaccharide (LPS) stimuli. In culture, M0, M1, and TCMs displayed distinct morphologies, in which M0 cells were smaller and rounded; M1 cells were larger, with protrusions giving a “ruffled” morphology that has been associated with macrophage activation^37^; and TCMs were elongated and spindle-like (Figure 2F). Differences in morphology were quantified based on the sphericities of the macrophages, in which M1 macrophages had higher sphericity indices than TCM macrophages (Figure 2G). Morphological changes in tumor macrophages *ex vivo* were investigated by two-photon microscopy using LLC tumor cells subcutaneously implanted into MacBlue;*CD11c*-mCherry mice. We found that the majority of visible myeloid cells were MacBlue+ macrophages, with only ∼15% of cells being *CD11c*-mCherry+ DCs and no MacBlue+*CD11c*-mCherry+ myeloid cells. MacBlue+ macrophages within treated tumors were more spherical than those in the control tumors, suggesting that they were morphologically similar to *in vitro* M1 macrophages (Figures 2H and 2I, Videos S8 and S9).

Video S8. Sphericity of TAMs in tumor treated with isotype control antibody, related to Figure 2H Live two-photon imaging of tumor-infiltrating TAMs (blue) and cDCs (red) in isotype control LLC tumor slices. Images were acquired for 15–20 min with 20-s time intervals, through a depth of 40 μm, and a maximum intensity projection is displayed. Scale bar represents 50 μm.

Video S9. Sphericity of TAMs in tumor treated with combo anti-PD1/PD-L1 antibodies, related to Figure 2H Live two-photon imaging of tumor infiltrating TAMs (blue) and cDCs (red) in combo-treated LLC tumor slices. Images were acquired for 15–20 min with 20-s time intervals, through a depth of 40 μm, and a maximum intensity projection is displayed. Scale bar represents 50 μm.

### TAMs and CD8^+^ T cells within checkpoint antibody-treated tumors display an activated phenotype

**Figure 3:**
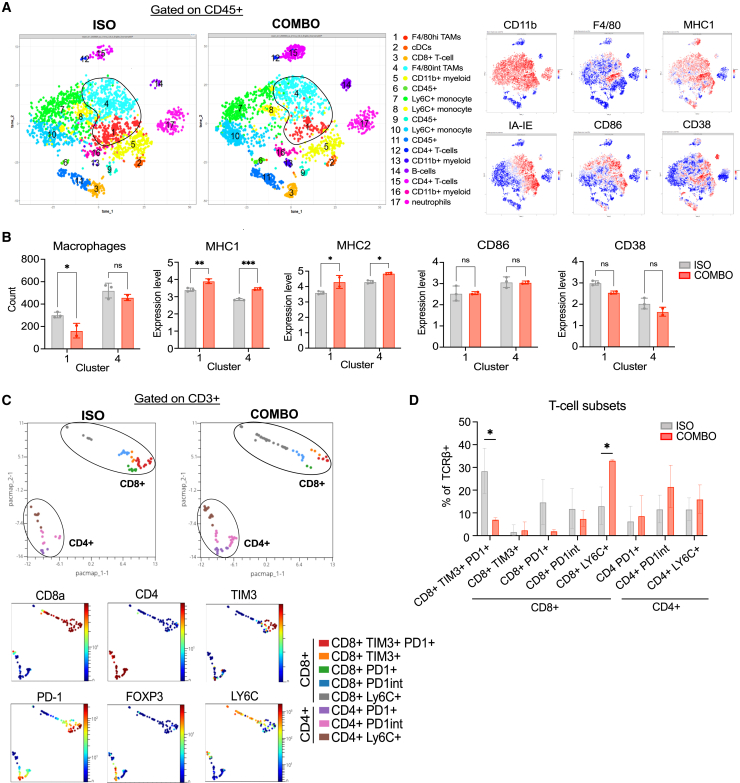
TAMs and CD8^+^ T cells within checkpoint antibody-treated tumors display an activated phenotype (A) Seventeen populations of CD45^+^ immune-infiltrating cells identified using tSNE at 15 dpi (left panels). CD11b, F4/80, MHC1, IA-IE, CD86, and CD38 fluorescence intensity plots (right panels). (B) Macrophage count and expression levels of MHC1, MHC2, CD86, and CD38 of Clusters 1 and 4. (C) Eight populations of CD3^+^ tumor-infiltrating T cells identified using PACMAP at 15 dpi (top panels). CD8a, CD4, TIM3, PD-1, FOXP3, and LY6C fluorescence intensity plots (bottom panels). (D) Frequencies of T cell subsets among CD3^+^ cells from isotype control and combo-treated tumors. Representative data of one CYTOF experiment containing *n* = 3 mice per experimental group and displayed as means ± SEMs. Analyzed by Student’s t test, one-way or two-way ANOVA; ∗*p* <0.05, ∗∗ *p* <0.01, ∗∗∗ *p* <0.001.

To further investigate how immune cells in the TME respond to ICB, we conducted high-dimensional analysis via cytometry time-of-flight (CYTOF) mass cytometry and identified 17 clusters of CD45^+^ immune cell infiltrates (Figure 3A, panel in Table S1). Within the 2 clusters of macrophages (Figure 3A), differential F4/80 expression delineated TAM clusters 1 and 4 (Figure 3B). We observed a significantly decreased count of cluster 1 (F4/80^hi^), but not cluster 4 (F4/80^int^), TAMs with combination treatment (Figure 3B). Both Cluster 1 and 4 TAMs increased expression of MHC1 and MHC2 with combination treatment, whereas no changes were observed in the costimulatory molecule CD86 or CD38, an ectoenzyme associated with immune cell activation (Figure 3B). Further analysis of CD3^+^ T cells identified 5 clusters of CD8^+^ and 3 clusters of CD4^+^ T cells (Figure 3C). Notably, treatment resulted in significant reductions in the frequencies of TIM3+ PD-1+ CD8^+^ T cells, characteristic of an exhausted T cell phenotype.^38^ Additionally, we observed significant increases in LY6C+ CD8^+^ T cells in treated tumors (Figure 3D), indicative of a stem-like or central memory CD8 T cell phenotype.^39^ Similar to the flow cytometric analyses, CYTOF did not reveal significant changes in CD4^+^ T cell subsets (Figure 3D). Thus, high-dimensional analysis further revealed that ICB specifically decreased the numbers of F4/80^hi^ TAMs, which was associated with the shifting of CD8^+^ T cell signatures from an exhausted toward a stem-like phenotype.

### TCMs respond to anti-PD-L1 by increasing pro-inflammatory features

**Figure 4:**
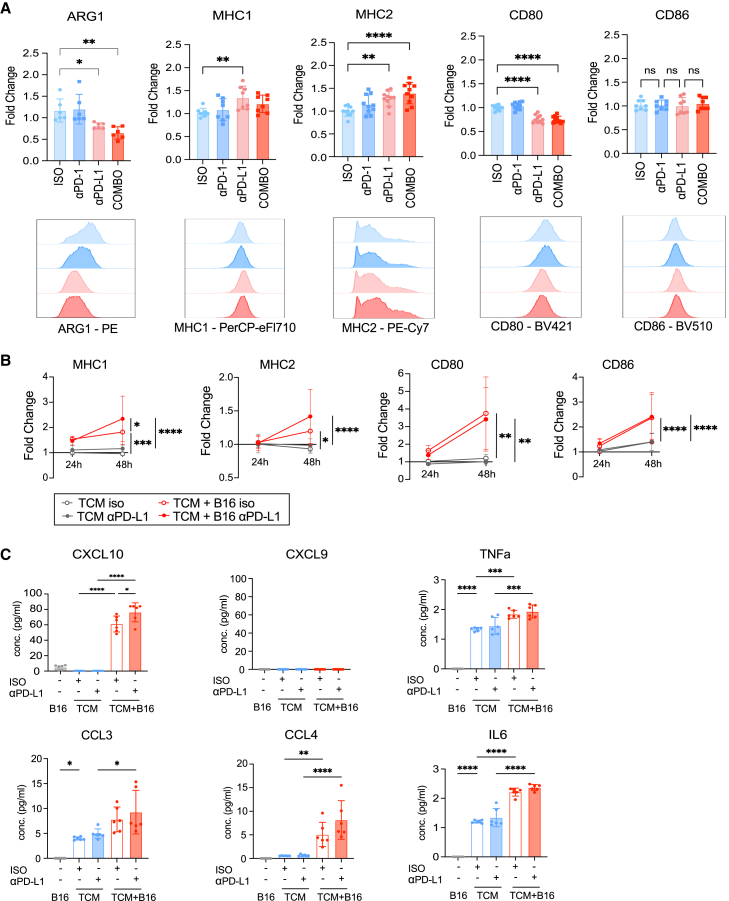
TCMs respond to anti-PD-L1 by increasing pro-inflammatory features (A) Fold change quantification and histograms of ARG1, MHC1, MHC2, CD80, and CD86 expression on TCMs treated with isotype control, anti-PD-1, anti-PD-L1, and combo treatment at 48 h. Data normalized to isotype control group of each marker. (B) Fold change quantification of MHC1, MHC2, CD80, and CD86 expression on TCMs co-cultured with B16-F10 tumor cells following treatment at 24 and 48 h. (C) Concentrations of CXCL10, CXCL9, tumor necrosis factor alpha, CCL3, CCL4, and IL-6 levels produced by TCMs quantified by LEGENDplex assay. Data shown represent combined data from 3 independent experiments with duplicate or triplicate wells and displayed as means ± SEMs. Analyzed by Student’s t test, one-way or two-way ANOVA; ∗*p* <0.05, ∗∗ *p* <0.01, ∗∗∗ *p* <0.001, ∗∗∗∗ *p* <0.0001.

*In vitro*-generated TCMs had upregulated PD-L1, but not PD-1, as compared to unpolarized M0 macrophages (Figure S3A). Similarly, TAMs isolated from B16-F10 tumors (Figure S3B) and the B16-F10 tumor cell line (Figure S3C) were PD-L1^hi^ and PD-1^lo^, suggesting that TAMs and B16 tumors were both responsive to anti-PD-L1 antibody. TCMs incubated in culture with anti-PD-L1 or a combination of anti-PD-1 and anti-PD-L1 decreased ARG1 expression and upregulated MHC1 and MHC2 expression (Figure 4A). We did not observe changes in the expression of costimulatory CD86, but CD80 expression was notably reduced by anti-PD-L1 or by combination anti-PD-1 and anti-PD-L1 treatment (Figure 4A). Incubation with anti-PD-1 alone did not drive changes in the expression of ARG1, MHC1/2, or CD80/86 (Figure 4A). TCMs co-cultured with B16-F10 tumor cells at a 1:10 ratio increased their relative expression of MHC1, MHC2, CD80, and CD86 over the course of 48 h as compared to TCMs cultured in medium alone (Figure 4B). Addition of anti-PD-L1 further increased MHC1 expression on these TCMs, with a slight but not statistically significant increase in MHC2 expression (Figure 4B). We replicated these experiments using LLC tumor cells, which confirmed similar changes occurred (Figure S3D). TCMs stimulated with LPS increased production of the CXCR3 ligand CXCL10, but not CXCL9, with anti-PD-L1 treatment (Figure 4C). The presence of tumor cells was sufficient to upregulate several other pro-inflammatory cytokines and chemokines, including tumor necrosis factor alpha, IL-6, chemokine C-C motif ligand 3 (CCL3), and CCL4, regardless of anti-PD-L1 treatment (Figure 4C). Similar trends were observed for cytokines produced at low levels, including vascular endothelial growth factor (VEGF), CCL2, IL-4, and IL-10 (Figure S3E). These findings indicate that anti-PD-L1 treatment can directly promote a pro-inflammatory signature in tumor macrophages.

### Anti-PD-L1 treatment increases phagocytic activity of TCMs

**Figure 5:**
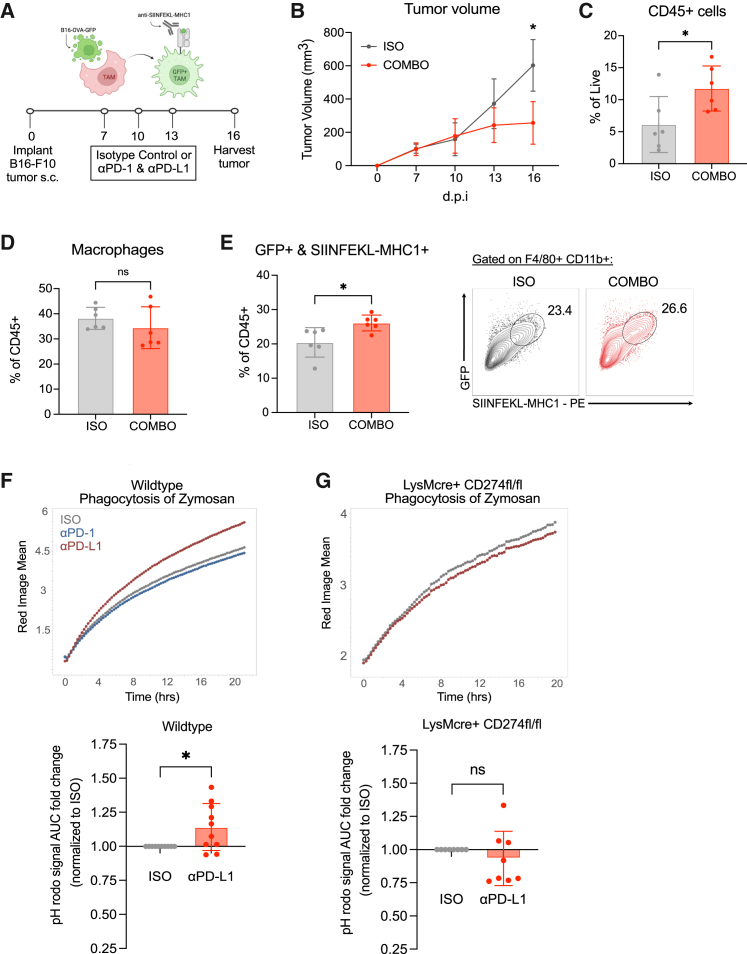
Anti-PD-L1 treatment increases phagocytic activity of TCMs (A) Experimental schematic of subcutaneous B16-OVA-GFP melanoma tumor mouse model treated with 3 doses of 200 μg of IgG2A isotype control or combination 200 μg anti-PD-1 and 200 μg anti-PD-L1 antibody intraperitoneally and harvested on day 16. (B) Tumor growth curves of isotype control and combo-treated mice. Data shown represent combined data from 2 experiments with *n* = 3 mice per experimental group. (C) Flow cytometric quantification of CD45^+^ immune cell infiltrate from isotype control and combo-treated tumors at 16 dpi. Each symbol represents a single tumor, and data are displayed as means ± SEMs. (D) Frequencies of TAMs from isotype control and combo-treated tumors at 16 dpi. (E) Frequencies of GFP+SIINFEKL-MHC1+ TAMs from isotype control and combo-treated tumors at 16 dpi (left panel). Representative flow cytometry plots of GFP and SIINFEKL-MHC1 within F4/80+CD11b+ TAM population (right panel). (F) Representative fluorescence curves of red pHrodo Zymosan particle intake by wild-type TCMs analyzed by Incucyte (top panel). Area under curve (AUC) fold change of red pHrodo signal normalized to isotype control wells (bottom panel). (G) Fluorescence curves of red pHrodo Zymosan particle intake by TCMs from *LysM*-Cre+ *Cd274*^fl/fl^ mice analyzed by Incucyte (top panel). AUC fold change of red pHrodo signal normalized to isotype control wells (bottom panel). Data shown represents combined data from 3 independent experiments with duplicate or triplicate wells and displayed as means ± SEMs. Analyzed by Student’s t test, one-way or two-way ANOVA; ∗ *p* <0.05.

Phagocytosis of tumor cells by TAMs is a potential mechanism for innate anti-cancer immunity^40^; therefore, we investigated TAM phagocytic activity against the B16-OVA-GFP murine melanoma cell line, which fluoresces green and expresses ovalbumin (OVA) neoantigen. As others have shown, phagocytosis of these tumor cells can be measured by ectopic GFP fluorescence, and processing and presentation of OVA peptides on MHC1 can be measured using anti-SIINFEKL-loaded MHC1 antibody (Figure 5A).^41^ Like B16-F10, subcutaneously implanted B16-OVA-GFP tumors in C57BL/6J mice treated with combination anti-PD-1 and anti-PD-L1 led to controlled tumor growth and increased CD45^+^ immune cell infiltration (Figures 5B and 5C). Although macrophage frequencies were unchanged (Figure 5D), combination anti-PD-1 and anti-PD-L1 led to a significantly increased proportions of GFP+ SIINFEKL-MHC1+ TAMs (Figure 5E), indicating that checkpoint antibody treatment promoted *in vivo* expansion of TAMs with increased phagocytic and antigen processing and presentation potential.

To further validate macrophage phagocytosis in real time, we quantified TCM engulfment of pHrodo Zymosan bioparticles, in which signal is generated upon exposure to the acidic phagolysosome environment.^42^ Anti-PD-L1-treated TCMs engulfed Zymosan particles more efficiently compared to those treated with isotype control (Figure 5F). The requirement for PD-L1 expression on macrophages to achieve this effect was tested using cells isolated from LysM-Cre;*Cd274*^fl/fl^ mice, in which Cre recombinase expression excises *Cd274*, the gene encoding PD-L1, in myeloid cells. We found that these PD-L1-deficient TCMs showed no change in bioparticle engulfment following anti-PD-L1 treatment (Figure 5G), indicating that PD-L1 ligation on macrophages by anti-PD-L1 was required for increased bioparticle engulfment.

### Anti-PD-L1-treated TCMs activate CD8^+^ T cells in an antigen-specific manner

**Figure 6:**
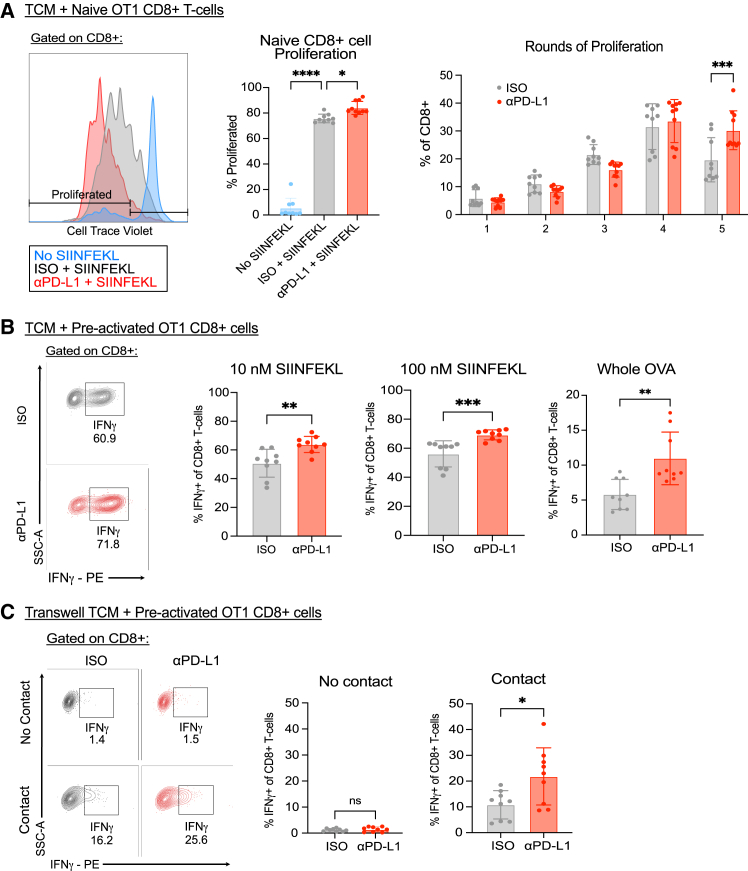
Anti-PDL1-treated TCMs activate CD8^+^ T cells in an antigen-specific manner (A) Histograms of CellTrace Violet proliferation peaks of naive OT1 CD8^+^ T cells co-cultured with isotype control or anti-PD-L1-treated TCMs pulsed with SIINFEKL peptide (left panel). Frequencies of proliferated naive CD8^+^ cells (middle panel), and rounds of proliferation of naive CD8^+^ T cells (right panel). (B) Representative flow cytometry plots (left panel) and frequencies of IFNγ+ pre-activated OT1 CD8^+^ T cells cultured with TCMs pulsed with 10 nM SIINFEKL, 100 nM SIINFEKL, or whole ovalbumin (right panels). (C) Representative flow cytometry plots (left panel) and frequencies of IFNγ+ pre-activated OT1 CD8^+^ T cells in contact with TCMs pulsed with 10 nM SIINFEKL or separated by 0.4-μm transwells (right panels). Data shown represent combined data from 3 independent experiments with triplicate wells and displayed as means ± SEMs. Analyzed by Student’s t test, one-way or two-way ANOVA; ∗∗∗∗ *p* <0.0001, ∗∗∗ *p* <0.001, ∗∗ *p* <0.01, ∗ *p* <0.05.

We investigated the effects of anti-PD-L1 on the ability of tumor macrophages to activate naive CD8^+^ T cells using OT1 TCR transgenic mice,^43^ in which T cells recognize the OVA peptide SIINFEKL presented within the context of MHC1. CellTrace Violet-labeled OT1 naive CD8^+^ T cells were co-cultured with SIINFEKL-pulsed TCMs that were previously treated with anti-PD-L1 or isotype control antibodies. Anti-PD-L1-treated TCMs were able to induce more proliferated CD8^+^ T cells and more rounds of proliferation than their control counterparts (Figure 6A, gating shown in S4A). In addition, we investigated anti-PD-L1-treated TCM reactivation of CD8^+^ T cells, as T cells are often activated in tumor-draining lymph nodes prior to trafficking to the tumor.^32^ Here, OT1 CD8^+^ T cells, activated by co-culturing splenocytes with SIINFEKL and IL-2 for 5 days, were subsequently co-cultured with antibody-treated TCMs that were pulsed with either SIINFEKL or whole OVA. Anti-PD-L1-treated TCMs induced significantly higher levels of IFNγ production among CD8^+^ T cells as compared to isotype control-treated TCMs (Figure 6B, gating shown in S4B). To assess whether CD8^+^ T cell activation was cell contact dependent, we utilized permeable membrane 0.4-μm transwells to physically separate TCMs and T cells. When CD8^+^ T cells were not in contact with TCMs, IFNγ production was lost, indicating that TCM reactivation of antigen-specific CD8^+^ T cells was contact dependent (Figure 6C). Thus, TCMs have the capacity to process and present antigens to activate CD8^+^ T cells, an effect augmented by anti-PD-L1 treatment.

### Macrophage repolarization and tumor control by dual PD-1/PD-L1 blockade is dependent on myeloid-specific PD-L1

**Figure 7:**
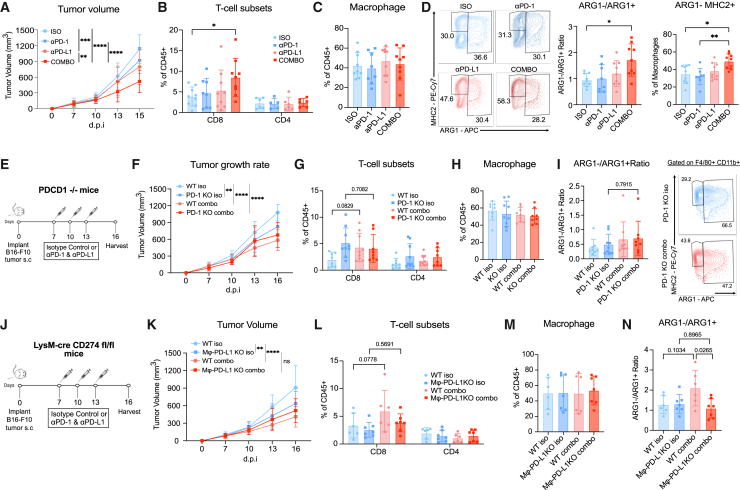
Macrophage repolarization and tumor control by dual PD-1/PD-L1 blockade is dependent on myeloid-specific PD-L1 (A) Tumor growth curves of isotype control, anti-PD-1, anti-PD-L1, and combo treatment of B16-F10 tumors. Data shown represent combined data from 3 experiments with *n* = 3 mice per experimental group. (B) Flow cytometric quantification of T cell subsets at 16 dpi. Each symbol represents a single tumor, and data are displayed as means ± SEMs. (C) Frequency of TAMs at 16 dpi. (D) Representative flow cytometry plots (left panel), ARG1-/ARG1+ ratio of TAMs (middle panel), and frequency of ARG1-MHC2+ TAMs (right panel) at 16 dpi. (E) Experimental schematic of subcutaneous B16-F10 melanoma tumor model in *Pdcd1*^−/−^ (PD-1 KO) mice treated with 3 doses of 200 μg IgG2A isotype control or combination 200 μg anti-PD-1 and 200 μg anti-PDL1 antibody intraperitoneally and harvested on day 16. (F) Tumor growth curves of WT and PD-1 KO mice. Data shown represent combined data from 3 experiments with *n* = 3 mice per experimental group. (G) Flow cytometric quantification of T cell subsets at 16 dpi. Each symbol represents a single tumor, and data are displayed as means ± SEMs. (H) Frequency of TAMs at 16 dpi. (I) Quantification of ARG1−/ARG1+ ratio of TAMs at 16 dpi (left panel). Representative flow cytometry plots of ARG1+ TAMs (left panel). (J) Experimental schematic of subcutaneous B16-F10 melanoma tumor model in LysM-Cre;*Cd274*^fl/fl^ mice treated with 3 doses of 200 μg of IgG2A isotype control or combination 200 μg anti-PD-1 and 200 μg anti-PD-L1 antibody intraperitoneally and harvested on day 16. (K) Tumor growth curves of WT and LysM-Cre;*Cd274*^fl/fl^ mice. Data shown represent combined data from 3 experiments with n = 2–3 mice per experimental group. (L) Flow cytometric quantification of T cell subsets at 16 dpi. Each symbol represents a single tumor, and data are displayed as means ± SEMs. (M) Frequency of TAMs at 16 dpi. (N) Frequency of ARG1−/ARG1+ ratio of TAMs at 16 dpi. Analyzed by Student’s t test, one-way or two-way ANOVA; ∗ *p* <0.05, ∗∗ *p* <0.01, ∗∗∗ *p* <0.001, ∗∗∗∗ *p* <0.0001.

Our efforts thus far indicated that anti-PD-L1 alone could affect tumor macrophage activation *in vitro*, yet our mouse tumor model was most responsive to checkpoint blockade when administered as a combination immunotherapy (Figure 1). We further dissected the contributions of the treatment components by comparing B16-F10 tumor growth in mice treated with anti-PD-1 or anti-PD-L1 monotherapies against combination and isotype control antibodies. Anti-PD-L1 treatment, but not anti-PD-1, significantly suppressed tumor growth, whereas the combination of both treatments achieved the most effective tumor growth control (Figure 7A). While both monotherapies slightly increased CD8^+^ T cell infiltration, only the combination-treated tumors achieved significantly increased frequencies of CD8^+^ T cell infiltration (Figure 7B). Although macrophage frequencies were similar (Figure 7C), we observed an increased ARG1−/ARG1+ macrophage ratio and overall ARG1− MHC2+ macrophage population in the combination-treated group compared to the monotherapy groups, (Figure 7D) suggesting anti-PD-1 and anti-PD-L1 therapy have synergistic effects in this regard.

We further examined the differential contributions of PD-1 and PD-L1 blockade using B16-F10 tumor cells implanted in *Pdcd1*^−/−^ mice (PD-1 knockoout [KO], Figure 7E), in which PD-1 receptor expression was genetically abrogated within the immune cells of the host. Our rationale was that because PD-1 ligation on CD8^+^ T cells has an impact on the TME (e.g., by increasing T cell effector cytokine secretion), it could activate TAMs in a manner independent of PD-L1:anti-PD-L1 antibody interactions. PD-1 KO mice treated with isotype controls exhibited significantly reduced tumor growth rates compared to their wild-type (WT) counterparts, confirming that abrogation of PD-1 itself was sufficient for anti-tumor immunity (Figure 7F). Both WT and PD-1 KO mice treated with combination immunotherapy also showed reduced tumor growth compared to WT controls (Figure 7F), indicating that PD-1 ligation by anti-PD-1 was not necessary for combination treatment efficacy. Combination treatment tended to increase CD8^+^ T cell infiltration in WT tumors, yet CD8^+^ T cell frequencies within tumors from PD-1 KO mice were unaffected (Figure 7G). Macrophage frequencies remained constant for all conditions (Figure 7H). Combination treatment led to an increased trend of ARG1−/ARG1+ TAM ratio in tumors from both WT and PD-1 KO mice. (Figure 7I), suggesting that blocking PD-L1 on TAMs alone was sufficient to induce an M1-like phenotype even in the absence of PD-1 signaling.

Since PD-L1 was highly expressed by both tumors and TAMs in our model (Figure S3), we utilized LysM-Cre;*Cd274*^fl/fl^ mice (Mφ-PD-L1 KO), in which myeloid cells lack PD-L1 expression (Figure 7J), to determine whether PD-L1 expression in macrophages was necessary for the macrophage repolarization observed with combination immunotherapy. Similar to PD-1 KO mice (Figure 7F), isotype-treated Mϕ-PD-L1 KO mice exhibited suppressed tumor growth compared to their *Cd274*^fl/fl^ (WT) littermate controls, and both combination-treated Mϕ-PD-L1 KO and WT mice had improved tumor control (Figure 7K). Combination treatment tended to increase CD8^+^ T cell infiltration in WT but not in Mϕ-PD-L1 KO tumors (Figure 7L). Macrophage frequencies remained constant for all conditions (Figure 7M), but interestingly, treatment of Mϕ-PD-L1 KO mice failed to increase the ARG1−/ARG1+ TAM ratio as seen in WT mice (Figure 7N), demonstrating that PD-L1 expression by TAMs was necessary for their remodeling after combination blockade treatment.

## Discussion

Although ICB immunotherapies have had a positive impact on improving cancer patient outcomes, it is still unclear why many patients do not respond to these treatments. Recent literature has revealed that the myeloid compartment, specifically TAMs, contribute to therapeutic resistance as they make up the majority of immune cell infiltrate in the tumor. TAMs play a key role in shaping the immunosuppressive TME by promoting tumor growth and suppressing the immune response. Recent work has indicated that tumor myeloid cells can respond to ICB and that this can shape clinical outcomes. However, the mechanics by which this operates is still under investigation; this information is necessary to reprogram myeloid cells to support anti-tumor immunity. In our study, we provide supportive evidence that TAMs are modified by ICB and further suggest that CD8^+^ T cell interactions with TAMs can be indicative of a therapeutically responsive TME.

Cytotoxic CD8^+^ T cells are essential during the anti-tumor response as they are the primary adaptive immune cell that function to recognize and clear tumor cells.^6^ T cells are initially primed in the tumor-draining lymph node prior to acquiring effector functions through interactions with APCs within the tumor.^32^ T cells not only need to efficiently infiltrate the tumor but also must make direct contacts with tumor cells for effective tumor clearance.^44^ CD8^+^ T cells located in the collagen-dense tumor stroma exhibit lower motility compared to those that infiltrate into the tumor core.^34^ However, previous studies have revealed that TAMs make long-lasting contacts, at least 20 min long, with tumor-infiltrating CD8^+^ T cells, which impede these T cells from infiltrating beyond the tumor stroma, preventing tumor clearance.^16^ Taken together, live-cell imaging data have supported a model in which CD8^+^ T cells are arrested within the TME, often in contact with immunosuppressive TAMs, where they accumulate exhaustion signatures.^41^

It follows that if TAM interactions are limiting T cell function, then these contacts must be abrogated to promote T cell infiltration. However, given that myeloid depletion clinical trials have had limited success,^19^ another possibility is that myeloid cells must persist within the tumor to support immune activation. Along these lines, anti-CTLA4 ICB therapy has been shown to increase T cell motility and enhance therapeutic efficacy by disrupting the stable interactions between T cells and tumor or myeloid cells.^45^ Similarly, in chronic viral infection models, which similarly drive T cell dysfunction, anti-PD1 therapy increases CD8^+^ T cell motility and activation.^46^ Our imaging data support this model, in that we visualized significant changes upon combination anti-PD-1/PD-L1 treatment, where CD8^+^ T cells display increased motility, which suggested improved surveillance of the TME. TAM densities remained consistent between treatment and control conditions. Interestingly, we showed that TAMs make contacts with CD8^+^ T cells with greater surface area overlap, which we hypothesize indicates a greater immune synapse activation of CD8^+^ T cells. To the best of our knowledge, our group is the first to show changes in the quality of TAM:CD8+ T cell contacts upon ICB treatment using live-cell two-photon microscopy. These large surface area contacts may facilitate more effective T cell:APC engagements and T cell activation. In support of this theory, we observed that treated tumor-infiltrating CD8^+^ T cells exhibited reduced path straightness, a characteristic of confined motility patterns and of activated T cells.^47^^,^^48^ These changes in interactions suggested that anti-PD-1/PD-L1 treatment may be promoting the activation of tumor-infiltrating CD8^+^ T cells by TAMs. This follows recent research highlighting the role of TAMs as important cross-presenting APCs within the TME,^41^ and our *in vitro* data corroborated the capacity of anti-PD-L1-treated macrophages to reactivate CD8^+^ T cells in an antigen-specific, cell contact-dependent manner. However, further research is needed to confirm the nature of these interactions *in vivo* and whether these stable contacts lead to enhanced T cell reactivation.

The blockade of PD-1 has been well researched and proven to be an effective treatment to restore T cell activation.^3^ However, the role of PD-L1 expression on TAMs remains controversial. *In vitro*, we observed highest upregulation of PD-L1 in M1-activating conditions, followed by TCMs. While a few recent studies suggest PD-L1^hi^ TAMs are immunostimulatory,^49^ most research on PD-L1-expressing TAMs has shown that they are suppressive and correlate with a poor prognosis in cancer patients.^50^^,^^51^ Investigations into the downstream signaling mechanisms of PD-L1 within myeloid cells have been limited, although studies in dendritic cells have indicated that intracellular or reverse PD-L1 signaling impacted migration.^52^ The intracellular signaling induced by PD-L1 blockade on TAMs, on the other hand, is still not well understood. In this study, we find that PD-L1 blockade promotes an activating phenotype in TAMs, and interestingly, this change can occur independently of IFNγ from T cells, which has been shown to be a major driver of pro-inflammatory macrophage polarization.^22^ These TAMs upregulate antigen presentation molecules MHC1 and MHC2, which are essential for T cell activation, and our *in vitro* studies confirm that anti-PD-L1-treated macrophages do become better T cell activators. Additionally, we found that blockade of the PD-L1 pathway, but not just the lack of PD-L1 expression, was necessary for TAM repolarization, supporting the idea that PD-L1 itself can participate in pro-inflammatory activation.

To further understand the effects of PD-L1 blockade on TAMs, future studies will focus on understanding the signaling mechanisms that drive these changes. Since NF-κB is a known pathway that leads to the activation of pro-inflammatory signaling pathways such as production of pro-inflammatory cytokines and chemokines,^53^ we hypothesized that PD-L1 blockade activates the NF-κB pathway in TAMs, shifting them toward a pro-inflammatory state. Activation of NF-κB can drive the production of chemokines such as CXCL10, which promotes CD8^+^ T cell infiltration and improves the anti-tumor response.^54^ Crosstalk between NF-κB and other pro-inflammatory signaling pathways may also be driven by the activation of STAT3. Intracellular PD-L1 was shown to promote tumor cell survival by inhibiting STAT3-dependent apoptosis,^55^ and ongoing work will investigate whether similar pathways are active within PD-L1-blocked TAMs. There are also possible mechanisms by which ICB is promoting TAM activation by blocking extracellular PD-L1 domain interactions. Recent work has shown that blocking PD-L1:CD80 interactions *in cis* promotes immunostimulatory functions in APCs.^56^^,^^57^ There are possibly multiple mechanisms at work during PD-L1 inhibition of myeloid cells that may be unique to the PD-L1 domain specificity of different therapeutic antibodies.

Our findings demonstrate that targeting PD-L1 on TAMs in combination with PD-1 blockade is a promising therapeutic combination to overcome ICB resistance and not redundantly targeting the same ICB pathway. Our data suggest that PD-L1 blockade reprograms TAMs and enhances their interactions with CD8^+^ T cells. Despite finding pro-inflammatory activation of TAMs, we found that PD-L1 blockade alone was not sufficient to control tumor growth *in vivo* and anti-PD-1 was still needed to promote the CD8^+^ T cell response and effective tumor control. Therefore, these results highlight the importance of combination therapies, which are necessary to activate CD8^+^ T cells while shifting TAMs toward an immunostimulatory state. In addition, further research is needed to determine whether the stable overlapping contacts we observed between TAMs and CD8^+^ T cells are sustained over time and lead to enhanced T cell reactivation. Ultimately, by understanding the mechanisms that drive PD-1:PD-L1 blockade therapeutic efficacy, more effective strategies can be developed to reprogram the TME, optimize combination therapies, and overcome ICB resistance to improve patient outcomes.

### Limitations of the study

In our treatment model, tumors were not completely eliminated, but growth rate was reduced within the immediate treatment phase. Other experimental systems are necessary to study repolarization of tumor macrophages over longer treatment regimens. This study of cellular mechanisms within the TME was conducted in the mouse model, and thus human relevance must be confirmed in future studies. Notably, anti-PD-1 is more often used in the clinic, although in mouse studies anti-PD-L1 has demonstrated efficacy. This suggests that mechanisms of immunosuppression and T cell reactivation may have some species specificity. Having established the phenotype of macrophage:CD8+ T cell interactions within the murine TME, future work must progress this understanding into human tumor immunology in order to rationally design immunotherapy regimens.

## Resource availability

### Lead contact

Requests for further information and resources should be directed to and will be fulfilled by the lead contact, Jessica Lancaster (lancaster.jessica@mayo.edu).

### Materials availability

This study did not generate new unique reagents.

### Data and code availability

• Primary data and research tools used in this study are available from the lead contact upon request. NanoString data discussed in this publication have been deposited in NCBI’s Gene Expression Omnibus (Edgar et al., 2002) and are accessible through GEO Series accession number GSE305308 (https://www.ncbi.nlm.nih.gov/geo/query/acc.cgi?acc=GSE305308).
• This paper does not report original code.
• Any additional information required to reanalyze the data reported in this paper is available from the lead contact upon request.

## Acknowledgments

The authors wish to thank Casey Ager (Mayo Clinic, Phoenix, AZ, USA) for insightful experimental guidance, Irene Adán-Barrientos (Immunobiology Laboratory, 10.13039/501100005884Centro Nacional de Investigaciones Cardiovasculares [CNIC], Spain) for protocol and assistance with pre-activated CD8^+^ T cell experiments, Caio Loureiro Salgado (Mayo Clinic, Phoenix, AZ, USA) for assistance with flow cytometry analysis, Igor Santiago Carvalho and Bruna de Gois Macedo (Mayo Clinic, Phoenix, AZ, USA) for supportive experimental knowledge, the Mayo Clinic Arizona Flow Cytometry Core Facility for assistance with flow cytometry, and the Mayo Clinic Immune Monitoring Core for assistance with CYTOF work and pilot funding. This research was supported by 10.13039/100000002NIH R01AG080037 (to J.N.L.). We also thank the Mayo Clinic David and Margaret Grohne Cancer Immunotherapy Program, the Mayo Clinic Breast Cancer SPORE, and the Mayo Clinic Hepatobiliary Cancer SPORE for pilot funding.

## Author contributions

J.N.L. and T.K. designed the experiments. T.K. performed the experiments. I.A.S.-J. assisted with flow cytometry experiments, and S.K performed supplemental experiments. T.K. analyzed the data. H.D. provided input for research design and interpretation. J.N.L. and T.K. wrote the manuscript. J.L., H.D., and T.K. reviewed and edited the manuscript.

## Declaration of interests

The authors declare no competing interests.

## STAR★Methods

### Key resources table

**Table:** 

REAGENT or RESOURCE	SOURCE	IDENTIFIER
**Antibodies**		

CD4 - BV480 (clone RM4-5)	BD Biosciences	Cat#565634; RRID:AB_2739312
CD8 - BUV395 (clone 53-6.7)	BD Biosciences	Cat#565968; RRID:AB_2739421
CD8 - AlexaFluor700 (clone 53-6.7)	Biolegend	Cat#100730; RRID:AB_493703
CD8 - PacBlue (clone 53-6.7)	Biolegend	Cat#100728; RRID:AB_493426
CD11b - AlexaFluor700 (clone M1/70)	Biolegend	Cat#101222; RRID:AB_493705
CD11c - BV711 (clone N418)	Biolegend	Cat#117349; RRID:AB_2563905
CD25 - PE (clone PC61)	Biolegend	Cat#102007; RRID:AB_312856
CD45 - PE-Cy5 (clone 30-F11)	Biolegend	Cat#103110; RRID:AB_312975
CD45 - BV510 (clone 30-F11)	Biolegend	Cat#110730; RRID:AB_1134168
CD86 - BV510 (clone GL-1)	Biolegend	Cat#105039; RRID:AB_2562370
CD80 - BV421(16-10A1)	Biolegend	Cat#104725; RRID:AB_10900989
ARG1 - APC (clone 14D2C43)	Biolegend	Cat#369706; RRID:AB_2814329
ARG1 – PE (clone W21047I)	Biolegend	Cat#165804
F4/80 - BV785 (clone BM8)	Biolegend	Cat#123141; RRID:AB_2563667
F4/80 – FITC (clone BM8)	Thermofisher	Cat#11-4801-85; RRID:AB_2637192
F4/80 – PE (clone BM8)	Biolegend	Cat#123110; RRID:AB_893486
FOXP3 - PE-CF594 (clone MF23)	BD Biosciences	Cat#562466; RRID:AB_11151905
GR1 - BV605 (clone RB6-8C5)	Biolegend	Cat#108439; RRID:AB_2562333
IFNgamma - PE (clone XMG1.2)	Biolegend	Cat#505808; RRID:AB_315402
Ly6C - APC-Cy7 (clone HK1.4)	Biolegend	Cat#128025; RRID:AB_10643867
MHC2 - PE-Cy7 (clone M5/114/15.2)	Biolegend	Cat#107630; RRID:AB_2069376
MHC1 - PerCP-eFl710 (clone AF6-88.5.5.3)	Thermofisher	Cat#46-5958-82; RRID:AB_2016714
PD-1 - BUV615 (clone J43)	BD Biosciences	Cat#752299; RRID:AB_2875816
PD-L1 - BUV737 (clone MIH5)	BD Biosciences	Cat#568361; RRID:AB_2688497
SIINFEKL-H2Kb - PE (clone 25-D1.16)	Biolegend	Cat#141604; RRID:AB_10895905
TCR-Valpha2 - PE (clone B20.1)	Biolegend	Cat#127808; RRID:AB_1134183
Armenian hamster anti-mouse TCRb (clone H57-597)	Thermofisher	Cat#14596182; RRID:AB_467758
Rabbit anti-mouse CD8 (clone S09-8E7)	Fisher Scientific	Cat#NB159216
Goat anti-Armenian Hamster IgG AF488	Thermofisher	Cat#A78963; RRID:AB_2925786
anti-rabbit IgG AF647	Thermofisher	Cat#A31573

**Chemicals, peptides, and recombinant proteins**		

Trypsin-EDTA	Thermofisher	Cat#25200056
Versene	Thermofisher	Cat#15040066
Matrigel Matrix	Corning	Cat#356237
Collagenase I	Worthington	Cat#LS004194
Collagenase IV	Worthington	Cat#LS004186
Anti-mouse IgG2a Isotype Control (clone C1.18.4)	BioXCell	Cat#BE0085
Anti-mouse PD-1 (clone RMP1-14)	BioXCell	Cat#BE0146
Anti-mouse PD-L1 (clone 10F.9G2)	BioXCell	Cat#BE0101
Recombinant murine M-CSF	Peprotech	Cat#315-02-100UG
Recombinant murine IL-10	Peprotech	Cat#210-10-10UG
Recombinant murine IL-4	Peprotech	Cat#214-14-100UG
Recombinant murine IFNg	Peprotech	Cat#315-05-100UG
OVA 257-264	GenScript	Cat#RP10611
Recombinant human IL-2	Peprotech	Cat#200-02-50UG
Ovalbumin protein full length	LSBio	Cat#LS-G132098-100
NuSieve GTG low-melting temperature agarose	Lonza	Cat#50080
HEPES	Sigma Aldrich	Cat#H3375-25G
Phenol red-free RPMI	Corning	Cat#50020PB
Sodium bicarbonate	Sigma Aldrich	Cat#S8875
CD45 microbeads mouse	Miltenyl Biotec	Cat#130-052-301
Zymosan pHrodo bioparticles	Sartorius	Cat#P35364
OCT compound	Sakura	Cat#4583
Prolong Gold anti-fade reagent	Thermofisher	Cat#P36930
DAPI	Thermofisher	Cat#D1306
RNAlater	Thermofisher	Cat#AM7020
Isopentane	Fisher Scientific	Cat#10030290
Matigel Matrix	Corning	Cat#CB40234A
LPS from E.coli	Sigma Aldrich	Cat#L4391
Zombie Red	Biolegend	Cat#423110
GhostDye Red 780	Tonbo Bioscience	Cat#13-0865-T100

**Critical commercial assays**		

FOXP3 Transcription factor Fix/Perm Kit	Thermofisher	Cat#00-5523-00
Cell Trace Violet Proliferation Kit	Thermofisher	Cat#C34557
Lengendplex Mouse Cytokine release syndrome panel	Biolegend	Cat#741024
EasySep Mouse Naïve CD8^+^ T Cell Isolation Kit	Stemcell	Cat#19858
EasySep™ Mouse TIL (CD45) Positive Selection Kit	Stemcell	Cat#100-0350
RNeasy Plus Universal Mini Kit	Qiagen	Cat#73404
Cytofix/Cytoperm Kit with GolgiPlug	BD Biosciences	Cat#BD555028

**Experimental models: Cell lines**		

Cancer cell line: B16-F10	ATCC	CRL-6475; RRID:CVCL_0159
Cancer cell line: B16-OVA-GFP	R. Vile (Mayo Clinic)	N/A
Cancer cell line: LLC	L. Rogers (Mayo Clinic)	RRID:CVCL_4358

**Experimental models: Organisms/strains**		

Mouse: C56BL/6J	Jackson Laboratories	Stock# 000664; RRID:IMSR_JAX:000664
Mouse: B6.SJL-Ptprca PepCb	Jackson Laboratories	Stock# 006584; RRID:IMSR_JAX:006584
Mouse: C57BL/6-Tg(TcraTcrb)1100Mjb/J	Jackson Laboratories	Stock# 003831; RRID:IMSR_JAX:003831
Mouse: B6.129-Lyzs/J (LysMcre)	Jackson Laboratories	Stock# 004781; RRID:IMSR_JAX:004781
Mouse: B6.Cg-*Cd274*^*tm3.1Shr*^/J (CD274 fl/fl)	Jackson Laboratories	Stock# 036255; RRID:IMSR_JAX:036255
Mouse: C57BL/6-Tg(CAG-EGFP)1Osb/J (CAG-EGFP)	Jackson Laboratories	Stock# 003291; RRID:IMSR_JAX:003291
Mouse: B6.Cg-Tg(CAG-DsRed∗MST)1Nagy/J (CAG-dsRed)	Jackson Laboratories	Stock# 006051; RRID:IMSR_JAX:006051
Mouse: MacBlue (CSF1R-CFP)	L. Ehrlich (U of Texas, Austin)	N/A
Mouse: CD11c-mCherry	L. Ehrlich (U of Texas, Austin)	N/A
Mouse: Pdcd1-/-	H. Kita (Mayo Clinic)	N/A
Mouse: MaFIA (CSF1R-EGFP)	Jackson Laboratories	JAX: 05070

**Software and algorithms**		

GraphPad Prism V.10.2.2	GraphPad	https://www.graphpad.com; RRID:SCR_002798
FlowJo V.10.10	TreeStar	https://www.flowjo.com; RRID:SCR_008520
Fiji-Image J	FIJI	https://imagej.net; RRID:SCR_002285
OMIQ	Insightful Science	https://www.omiq.ai
Imaris	Bitplane	https://imaris.oxinst.com; RRID:SCR_007370
Cytek SpectroFlo	Cytek Biosciences	RRID:SCR_025494
Rosalind	Rosalind	https://www.rosalind.bio

### Experimental model and study participant details

C57BL6/6J (JAX #000664), B6.SJL-Ptprca PepCb (CD45.1; JAX #006584), C57BL/6-Tg(TcraTcrb)1100Mjb/J (OT-I^58^; JAX #003831), CAG-EGFP^59^ (JAX #003291), CAG-dsRed^60^ (JAX #006051), *Csf1r*-EGFP (MaFIA,^61^ JAX #005070), LysM-Cre^62^ (JAX #004781), and *Cd274*^fl/fl^^63^ (JAX #036255) mice were purchased from Jackson Laboratories. MacBlue^64^ (CSF1R-CFP) and *CD11c*-mCherry^65^ mice were gifted by L. Ehrlich (University of Texas at Austin). *Pdcd1*^-/-^ mice^66^ were gifted by H. Kita (Mayo Clinic). Mice were bred and maintained under specific-pathogen free conditions with controlled room temperature and 12-hour light / 12-hour dark day cycle in the Mayo Clinic animal facility. Male mice 6-10 weeks of age were used in experiments in accordance with protocols approved by the Mayo Clinic Institutional Animal Care and Use Committee (AUP #A00005857-21-R24).

B16-F10 murine melanoma tumor cells (ATCC #CRL-6475), Lewis lung carcinoma (LLC) murine tumor cells (gifted by L. Rogers, Mayo Clinic), and B16-GFP-OVA murine melanoma tumor cells (gifted by R. Vile, Mayo Clinic) were cultured under normoxic conditions at 37°C, 5% CO_2_ in Dulbecco's Modified Eagle's Medium (DMEM; Thermofisher), 10% fetal bovine serum (FBS; Thermofisher), 1% Penicillin-streptomycin (Thermofisher).

### Method details

#### *In vivo* tumor mouse model

Tumor cells were grown to 80% confluency, harvested with 0.05% Trypsin-EDTA (Thermofisher), and resuspended in phosphate buffered saline (PBS). 2.5 x 10^5^ B16-F10 cells or 1 x 10^6^ LLC cells were aliquoted and resuspended in a 1:1 ratio of PBS and Matrigel Matrix (Corning) in a final volume of 50 μl and loaded into 0.5 ml 28G insulin syringes (BD). Tumors were injected subcutaneously into the right flank of anesthetized mice and were allowed to grow until palpable ∼80 mm^3^ in volume. Mice were treated with 200 μg of IgG2a isotype control (C1.18.4, BioXCell InVivoMAb), anti-PD-1 (RMP1-14, BioXCell InVivoMAb), and/or anti-PD-L1 (10F.9G2, BioXCell InVivoMab) in 50 ul of PBS and injected intraperitoneally three times every 3 days. Tumor volume was measured on treatment days using a digital caliper and calculated according to the formula: tumor volume = (width^2^ ∗ length)/2.

#### Immunofluorescence

Harvested tumors were submerged in Optimal Cutting Temperature (OCT) compound (Sakura) in 37 x 24 x 10 μm cryomolds (VWR) and frozen on top of dry ice and isopentane (Fisher Scientific). Frozen tissue was cut into 10-30-um thin sections using a HM525NX cryostat (Thermo Scientific) on poly-L-lysine coated microscope slides (VWR). Tissues were fixed in cold acetone for 20 mins and outlined with a PAP pen, then washed with 0.1% Tween-20 in PBS between each following step. Slides were blocked with 2% bovine serum albumin fraction V (BSA) in PBS for 20 mins, stained at 4°C overnight with primary antibodies in blocking buffer: Hamster anti-mouse TCRβ (H57-597, 1:200, Invitrogen), anti-mouse CD8 (53-6.7, 1:100, BD), F4/80 (BM8, 1:200, Biolegend). Secondary antibodies prepared in blocking buffer were stained for 1 hour at room temperature: AF488 goat anti-hamster IgG (1:200, Jackson), AF647 donkey anti-rat IgG (1:200, Thermofisher). Nuclei were then stained with 3uM of DAPI (Thermofisher) for 10 mins at room temperature and coverslips sealed with Prolong Gold anti-fade reagent (Thermofisher). Sections were imaged using a LSM 800 confocal microscope (Zeiss). Fluorescence of CD8, TCRβ, F4/80, and DAPI were quantified using Imaris (v10, Bitplane).

#### Flow cytometry

Tumors were harvested on day 16 and enzymatically digested using 1 mg/ml of Collagenase I and IV (Worthington) for 20 min at 37°C, washed with FWB (1x PBS + 2% fetal bovine serum), then mechanically digested through a 40-um cell strainer into a single cell suspension. Cells were stained with fluorochrome-conjugated antibodies for 20 min at 4°C, then washed with FWB. For intracellular staining, cells were fixed for 20 min at 4°C using the FOXP3 Fixation/Permeabilization kit (Thermofisher) as per manufacturer’s instructions then washed in permeabilization buffer. Cells were then stained with fluorochrome-conjugated intracellular antibodies for 20 min at 4°C, washed, and resuspended in PBS. Samples were read on a BD Symphony cytometer and data was analyzed using FlowJo software (v10, Treestar).

#### Generation of bone-marrow derived macrophages (BMDM)

Bone-marrow from the tibia and femur of C57BL/6J or LysM-Cre;*Cd274*^fl/fl^ mice were flushed out with PBS and cultured in 10-cm petri dishes with 10 ml of DMEM supplemented with FBS (10%), Penicillin-Streptomycin (1%), and 20 ng/ml of M-CSF (Peprotech) for 5 days. Fresh media was topped up every 2-3 days. M0 macrophages were cultured in DMEM supplemented with M-CSF (20 ng/ml). Tumor-conditioned macrophages (TCMs) were generated by culturing macrophages in DMEM supplemented with M-CSF (20 ng/ml) and 20 ng/ml of IL-4 and IL-10 (Peprotech), and B16-F10 or LLC tumor conditioned media. M1 macrophages were generated by culturing macrophages in DMEM supplemented with M-CSF (20 ng/ml, Peprotech), 10 ng/ml of LPS (Sigma Alrich), and 20 ng/ml of IFNg (Peprotech). Macrophages were cultured for 48 hours in 6-well non-treated plates and phase images were taken on EVOS FL Auto Imaging system (Life Technologies). Cells were detached with Versene (Thermofisher), washed with PBS, then stained for downstream flow cytometry analysis.

#### Two-photon microscopy

CD8^+^ T-cells were enriched from OT1-EGFP or OTI-dsRed mice spleen and lymph nodes using EasySep Mouse Naïve CD8^+^ T-cell isolation kit (Stem Cell) as per manufacturer’s instructions. 1 x 10^6^ cells in 100 μl of PBS were retro-orbitally injected into MacBlue;CD11c-mCherry, MacBlue, or *Csf1r*-EGFP mice 24 hours prior to imaging. Tumors were dissected and embedded in 4% NuSieve GTG low-melting temperature agarose (Lonza) in PBS at 37°C. Tumors were sectioned into 300-400μm thick slices using a VT1200S Microtome (Leica) in cold PBS, with speed 0.20 mm/s and amplitude at 0.6 mm. Slices were collected in complete RPMI (cRPMI), consisting of FBS (10%), Penicillin-Streptomycin (1%), Non-essential Amino Acids (1%), Sodium Pyruvate (1%), GlutaMAX (1%), Beta-mercaptoethanol (0.1%), and kept on ice until imaging. Tumor slices were transferred and secured in an imaging chamber (Harvard Apparatus) on the microscope stage. Perfusion medium, consisting of phenol red-free RPMI (Corning) supplemented with 2 g L^−1^ sodium bicarbonate (Sigma Aldrich) and 5 mM HEPES (Sigma Aldrich) was circulated through the imaging chamber using a micro-perfusion high-flow pump (Bioptechs). The perfusion medium was aerated with 95% O_2_/5% CO_2_ and maintained at 37°C with a heated microscope stage and inline perfusion heater (Harvard Apparatus). Images were acquired every 20s, through a depth of 40 μm, at 5-μm intervals for durations of 15–20 min, using an Investigator microscope (Bruker) with a 20x water immersion objective (Olympus, NA 1.0) and PrairieView software (v.5.5, Bruker). The sample was illuminated with a Chameleon Discovery NX ultrafast pulsed laser (Coherent) tuned to 840 nm (for MacBlue) or 920 nm (for EGFP) with a fixed line at 1040 nm (for mCherry/dsRed). Emitted light was passed through 460/50, 525/50, and 595/50 band-pass filters (Chroma) to separate GaAsP detectors for detection of CFP (blue), EGFP (green) and mCherry/dsRed (red) fluorescence, respectively. Migratory paths for T cells were tracked, and mean cell velocity and path straightness for each cell calculated using Imaris (v10, Bitplane). Distance Transformation function in ImarisXT was used to calculate distances between T cells and TAMs. Sphericity function in ImarisXT was used to calculate sphericity of TAMs. Cell surface area overlap was calculated using ImarisXT software overlapped volume statistic. 2-photon imaging was also conducted from lymph node slices, as conducted previously, from naïve MacBlue mice in which OTI-EGFP CD8^+^ naïve T cells were adoptively transferred.^67^

#### CYTOF

B16-F10 tumors grown in C57BL6/J mice were treated with anti-PD-1 and anti-PD-L1 as previously described. Tumors were enzymatically digested with Collagenase I and IV (Worthington) and CD45- cells were depleted using CD45 microbeads (Miltenyl Biotec) and LD Columns (Miltenyl Biotec). CD45^+^ enriched cells were stained with metal-conjugated antibodies and analyzed by Mass Cytometry-Helios CyTOF at the Mayo Clinic Arizona Immune Monitoring Core. Data was analyzed using OMIQ software.

#### *In vitro* tumor-conditioned macrophage and tumor co-culture

TCMs and B16-F10 tumor cells were co-cultured at a 1:0 and 1:10 ratio (3 x 10^4^ TCMs:3 x 10^5^ B16-F10 cells) with 20 μg mL^-1^ of isotype control, anti-PD-1 and/or anti-PD-L1 antibodies for 24 or 48 hours in 6-well non-treated plates. Macrophages were detached with Versene (Thermofisher) and stained for surface and intracellular markers, then read on a BD Symphony cytometer. For cytokine analysis, LPS (10 ng mL^-1^; Sigma Aldrich) was added into the culture and supernatant was collected at 24 hours and stained using the LEGENDplex Mouse Cytokine Release Syndrome Panel kit (Biolegend) as per manufacturer’s instructions and run on a BD Symphony cytometer.

#### Phagocytosis assays

5 x 10^3^ TCMs in 50 uL of DMEM supplemented with 20 ng mL^-1^ of M-CSF (Peprotech) were plated in a 96-well flat bottom plate and allowed to adhere for 2 hours. 20 μg mL^-1^ of isotype control or anti-PD-L1 in 50 μL of DMEM was added per well and incubated at 37°C for 24 hours. 5 μg of red Zymosan pHrodo bioparticles (Sartorius) were added to each well and placed in an Incucyte incubator (Sartorius), scanned every 15 mins for 24 hours at 10x magnification. For the *in vivo* phagocytosis analysis, B16-OVA-GFP tumors were grown in C57BL6/J mice and treated with 200 μg isotype control, or anti-PD-1 and anti-PD-L1 antibodies as previously described for the B16-F10 tumor treatment timeline. Tumors were harvested and prepared for flow analysis as previously described.

#### Naïve T-cell and tumor-conditioned macrophage co-culture

Inguinal lymph nodes and spleens were isolated from OT1 mice and mechanically digested through a 100-μm cell strainer into a single cell suspension. Cells were enriched for naïve CD8^+^ T-cells using the Easy Sep naïve CD8^+^ T-cell kit (Stemcell) as per manufacturer’s instructions. Enriched naïve OT1 T-cell were stained with CellTrace Violet (Thermofisher) in PBS for 15 minutes and washed with cRPMI. Tumor-conditioned macrophages were generated as previously described and treated with 20 μg mL^-1^ of IgG2a isotype control, anti-PD-1 and/or anti-PD-L1 antibodies in 6-well non-treated plates for 24 hours. TCMs were then pulsed with 10 nM SIINFEKL (257-264, GenScript) for 30 minutes at 37°C and washed with DMEM. TCMs were co-cultured with OT1 cells in a 96-well U-bottom plate at a 1:5 ratio (20,000 TCMs:100,000 OT1 cells) in cRPMI supplemented with 10 ng mL^-1^ of IL-2 (40 ng mL^-1^, Peprotech) for 72 hours. Cells were collected and washed with FWB, stained for cell surface markers, and read on a BD Symphony cytometer.

#### Pre-activated OT1 and tumor-conditioned macrophage co-culture assay

OT1 splenocytes were cultured in cRPMI supplemented with IL-2 (10 ng mL^-1^, Peprotech) and OVA SIINFEKL (257-264, 10nM or 100nM, GenScript) or full-length ovalbumin protein (1 mg mL^-1^, LSBio) for 5 days. TCMs were generated as described and treated with 20 μg mL^-1^ of isotype control, anti-PD-1 and/or anti-PD-L1 for 24 hours. TCMs and OT1 cells were co-cultured in a 1:5 ratio (20,000 TCMs:100,000 OT1 cells) in a 96-well U-bottom plate in cRPMI. After 1 hour, Golgistop protein transport inhibitor (BD) was added in the culture for 2 hours then cells were collected and stained for IFNg (XMG1.2, Biolegend) and fixed with the Cytofix/Cytoperm plus fixation/permeabilization kit (BD) as per manufacturer’s instructions and read on a BD Symphony cytometer. Transwell experiments were conducted using 0.4-um inserts in a 24-well plate (Corning).

#### Nanostring

B16-F10 tumors grown in C57BL6/J mice were treated with anti-PD-1 and anti-PD-L1 as previously described. Harvested tumors were directly submerged in RNAlater (Thermofisher) until ready to process. Tumor samples were sonicated prior to RNA extraction and processed using the RNAeasy Plus Mini kit (Qiagen) as per manufacturer’s instructions. Samples were sequenced using Nanostring’s Tumor Signaling 360 Panel and run on the nCounter Pro Analysis System. Analysis was conducted using Rosalind (Nanostring).

### Quantification and statistical analysis

Data in this study are presented as means ± SEM for n = 3–842 representing the number of mice, cells, or technical replicates depending on the assay performed. Statistical analysis were performed using GraphPad Prism software and can be found in the corresponding figures and figure legends. Depending on the dataset, unpaired t-tests, one-way ANOVA, or two-way ANOVA tests were performed. P-values <0.05 were considered statistically significant. Significant values are represented as ∗ P<0.05, ∗∗ P<0.01 and ∗∗∗ P<0.001.
