# Supplementary material for: Macrophage repolarization by immune checkpoint blockade drives T cell engagement in the tumor microenvironment
Source: iScience. 2025 Sep 10;28(10):113538. doi: 10.1016/j.isci.2025.113538 (PMC12494938; doi:10.1016/j.isci.2025.113538)
Supplement: Document S1. Figures S1–S4 [file mmc1.pdf]

iScience, Volume *28*

Supplemental information

Macrophage repolarization by immune checkpoint blockade drives T cell engagement in the tumor microenvironment

Tina Kwok, Ildefonso A. Silva-Junior, Sara Korpe, Haidong Dong, and Jessica N. Lancaster

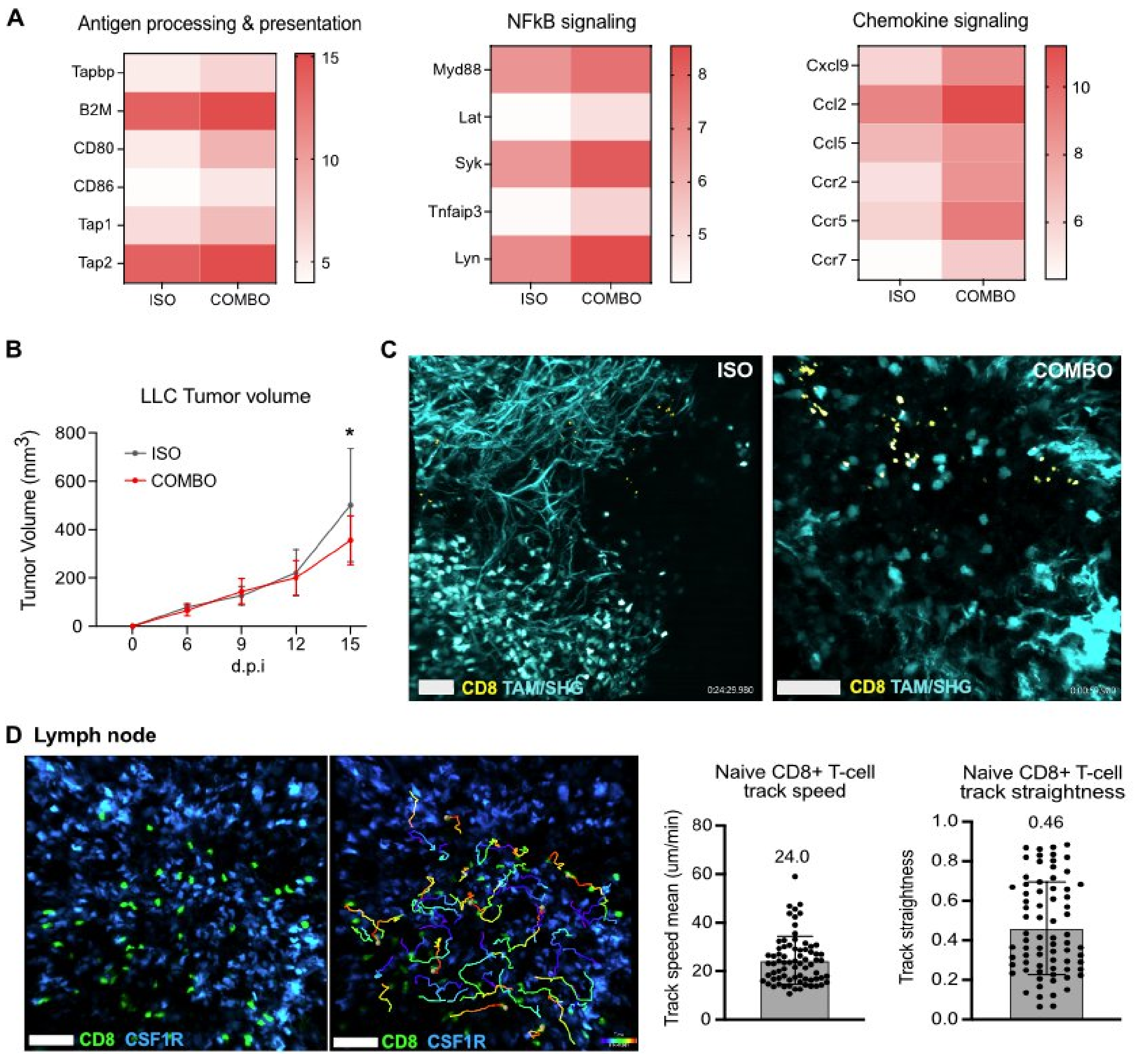

Figure S1. Related to Figure 1. Checkpoint antibody-treated tumors promote a pro-inflammatory tumor microenvironment

(A) CD45+ immune infiltrate cells isolated from isotype control and combo treated B16-F10 tumors were sequenced using Nanostring's Tumor Signaling 360 panel. Heatmaps of antigen processing and presentation (left panel), NFkB signaling (middle panel) and chemokine signaling (right panel) genes shown. **(B)** Tumor growth curve of Lewis lung carcinoma (LLC) subcutaneous tumors in MacBlue;CD11c-mCherry reporter mice treated with three doses of 200 mg IgG2A isotype control, or combination 200 mg anti-PD-1 and 200 mg anti-PD-L1 antibody intraperitoneally, and harvested on day 15. **(C)** Live two-photon images of isotype control and combo treated LLC tumors from MacBlue mice (blue macrophages) with adoptively transferred dsRed-CD8+ T-cells (yellow). Second harmonic generation (SHG) in blue. Scale bar represents 50 mm. **(D)** Live two-photon images of ex vivo lymph node slice from Macblue mice with adoptively transferred GFP-CD8+ T-cells (green). Quantification of naïve CD8+ T-cell track speed mean and straightness. Scale bar represents 50 mm.

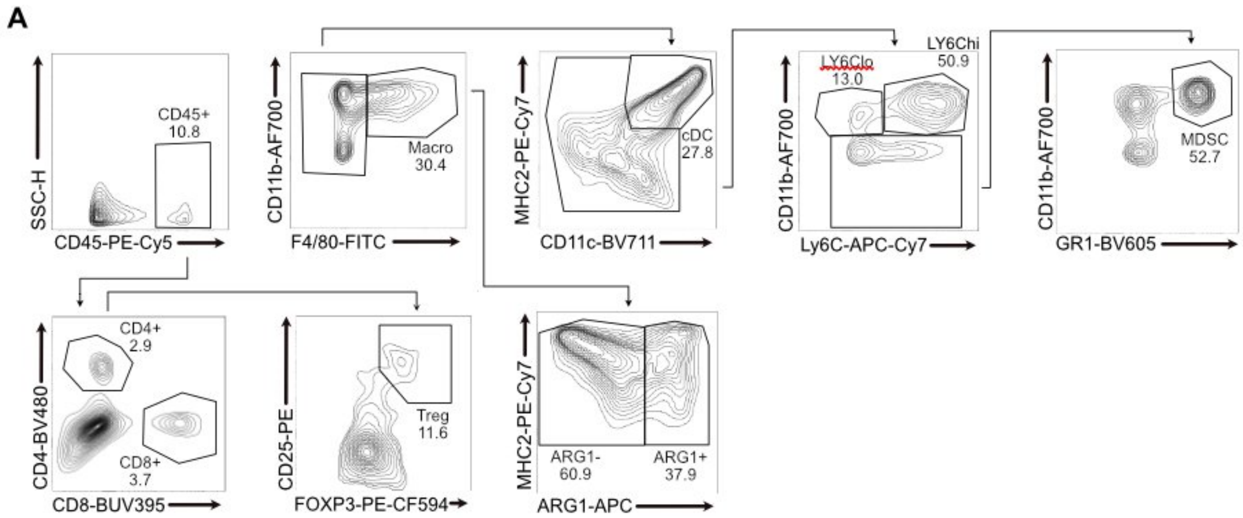

Figure S2. Related to Figure 2. Flow cytometry gating of tumor immune cell infiltrate
(A) Flow cytometry gating strategy of immune infiltrate from treated B16-F10 tumors; CD45+ immune cells, CD11b+F4/80+ TAMs to distinguish ARG1+ and ARG1- TAMs, CD11c+MHC2+ cDCs, CD11b+LY6C+ monocytes, CD11b+GR1+ MDSCs, CD8+ and CD4+ T-cells, and CD25+FOXP3+ Tregs.

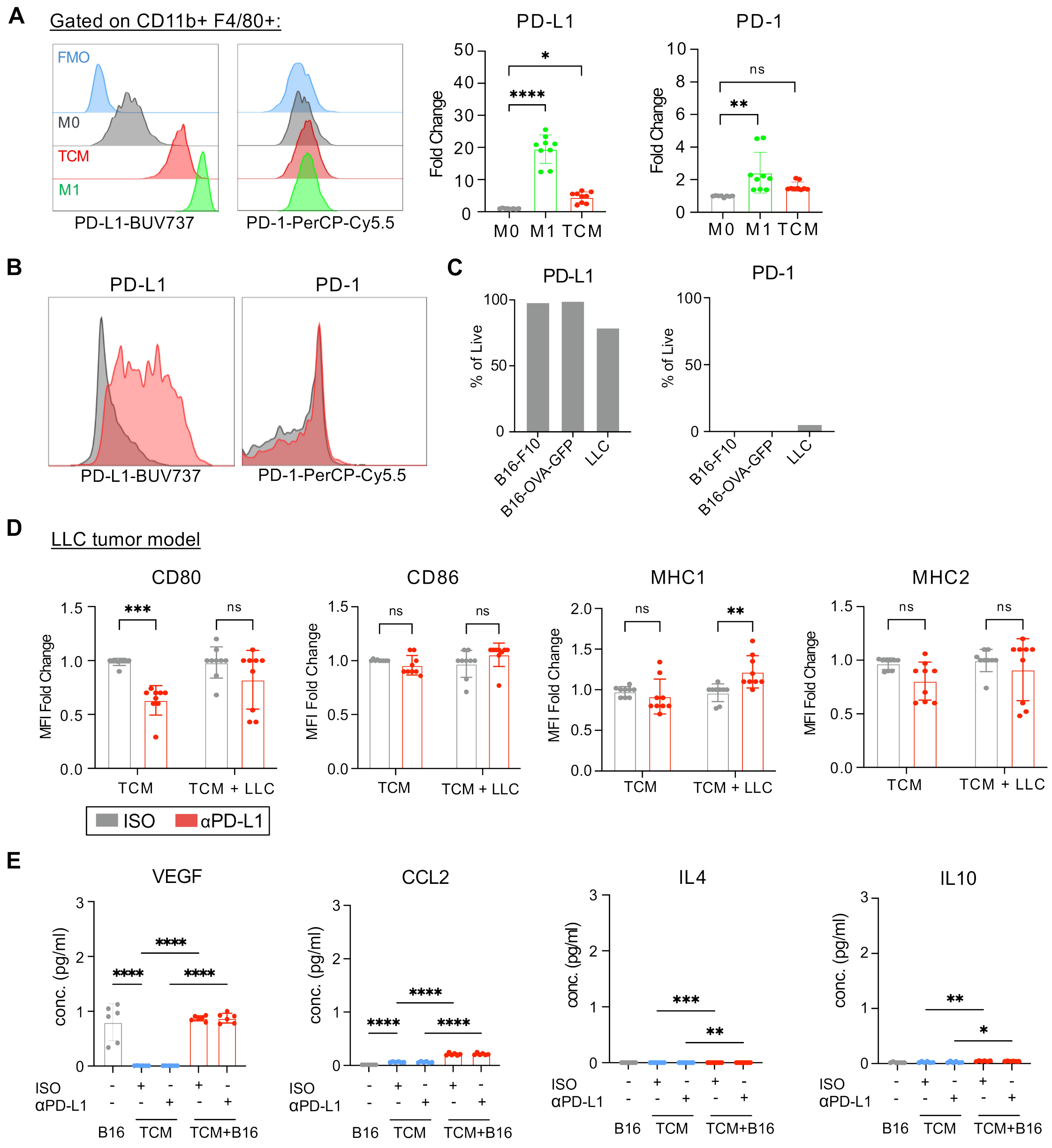

Figure S3. Related to Figure 4. In vitro generated TCMs resemble TAMs found in mouse B16-F10 tumors

(A) Flow cytometry of M0 (grey), M1 (green), and TCM (red) cells stained for PD-1 and PD-L1 expression. **(B)** Flow cytometry of TAMs from B16-F10 tumors (red) stained for PD-1 and PD-L1 expression. Grey represents fluorescence minus one (FMO) of each marker. **(C)** Flow cytometry of B16-F10, B16-OVA-GFP, and LLC tumor cell lines stained for PD-1 and PD-L1 expression **(D)** TCMs were cultured alone or in a 1:10 ratio with LLC tumor cells for 24 and 48 hours and stained for MHC1, MHC2, CD80, and CD86 expression**.** **(E)** Concentration levels of VEGF, CCL2, IL-4, and IL-10 produced by TCMs quantified by Legendplex cytokine analysis kit. Data shown represents combined data from 3-4 independent experiments. Analyzed by Student's t-test, one-way or two-way ANOVA, p: **** < 0.001, ** < 0.01, * < 0.05.

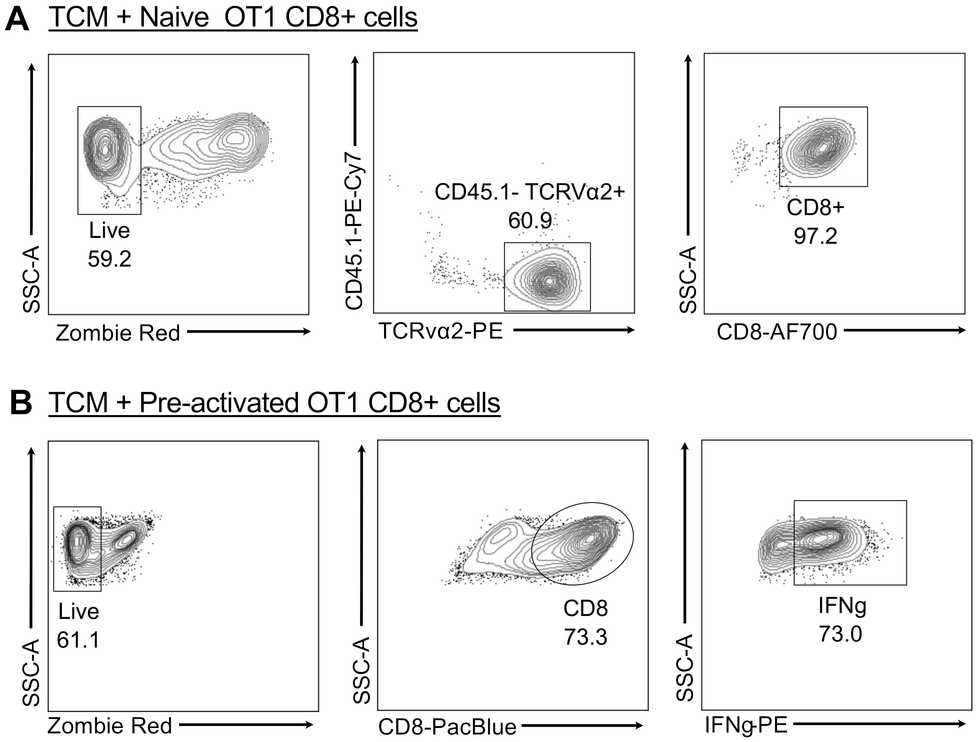

Figure S4. Related to Figure 6. Flow cytometry gating strategy of CD8+ T-cells
(A) Gating strategy for CD8+ T-cells and **(B)** IFNg+CD8+ T-cells from in vitro activation assays.

ms_TME_Panel_02

No.	Sp.	Experiment Name	Label	Target	Clone	Location	Amount Per Test (μL)
1	Ms	mCART_TME_Panel_02	089Y	CD45	30-F11	Surface	0.50
5	Ms	Ms_T-cell_Panel_01	141Pr	CD39	24DMS1	Surface	1.00
6	Ms	mCART_TME_Panel_02	142Nd	CD11c	N418	Surface	1.00
7	Ms	Ms_T-cell_Panel_01	143Nd	TCRb	H57-597	Surface	**0.50**
8	Ms	mCART_TME_Panel_02	144Nd	MHC Class I	28-14-8	Surface	0.50
9	Ms	mCART_TME_Panel_02	145Nd	CD4	RM4-5	Surface	0.50
10	Ms	mCART_TME_Panel_02	146Nd	F4/80	BM8	Surface	0.75
11	Ms	mCART_TME_Panel_02	147Sm	IDO		Intracellular/Nuclear	1.00
12	Ms	Ms_T-cell_Panel_01	149Sm	CD366 (Tim-3)	RMT3-23	Surface	1.00
13	Ms	mCART_TME_Panel_02	151Eu	CD86	GL1	Surface	1.00
14	Ms	mCART_TME_Panel_02	152Sm	CD3e	145-2C11	Surface	**0.50**
15	Ms	mCART_TME_Panel_02	153Eu	PD-L2	TY-25	Surface	1.00
16	Ms	mCART_TME_Panel_02	154Sm	IFNα1	F18	Intracellular/Nuclear	1.00
17	Ms	mCART_TME_Panel_02	155Gd	HVEM	2024B	Intracellular/Nuclear	1.00
18	Ms	mCART_TME_Panel_02	156Gd	Galectin 9	Polyclonal	Intracellular/Nuclear	1.00
19	Ms	Ms_T-cell_Panel_01	158Gd	FoxP3	FJK-16s	Intracellular/Nuclear	1.00
20	Ms	Ms_T-cell_Panel_01	159Tb	CD279 (PD-1)	RMP1-30	Surface	1.00
21	Ms	mCART_TME_Panel_02	161Dy	Ly-6G	1A8	Surface	0.25
22	Ms	mCART_TME_Panel_02	164Dy	IFNβ	MIB-5E9.1	Intracellular/Nuclear	1.00
23	Ms	Ms_T-cell_Panel_01	165Ho	Thy1.1		Surface	0.50
24	Ms	mCART_TME_Panel_02	166Er	CD19	6D5	Surface	0.50
25	Ms	Ms_T-cell_Panel_01	167Er	CD38	90	Surface	1.00
26	Ms	mCART_TME_Panel_02	168Er	CD8a	53-6.7	Surface	0.25
27	Ms	mCART_TME_Panel_02	169Tm	CD274 (PD-L1)	10F.9G2	Surface	1.00
28	Ms	mCART_TME_Panel_02	170Er	CD161 (NK1.1)	PK136	Surface	0.50
29	Ms	mCART_TME_Panel_02	171Er	CD11b (Mac-1)	M1/70	Surface	0.25
30	Ms	mCART_TME_Panel_02	174Yb	EGFRvIII	L8A4	Surface	2.00
31	Ms	mCART_TME_Panel_02	175Yb	Ly-6C	HK1.4	Surface	0.10
32	Ms	mCART_TME_Panel_02	176Yb	FasL	MFL3	Surface	1.00
33	Ms	mCART_TME_Panel_02	209Bi	I-A/I-E	M5/114.15.2	Surface	0.10

Table S1. Related to Figure 3. CYTOF panel for B16-F10 immune infiltrate
List of targets from the mouse tumor microenvironment CYTOF panel created by the Mayo Clinic Immune Monitoring Core.
